# Slower motor speed as a predictor of suicide attempts in high-risk youth

**DOI:** 10.1017/S0033291726103675

**Published:** 2026-05-05

**Authors:** Meilin Jia-Richards, Sriram Ksheeraja, Sarah Riston, Elijah Goodfriend, Adam Z. Melhem, Katherine Recker-Mohn, Veda Murthy, Andrew Luskin, Nermin Toukhy, Kehui Chen, Antoine Douaihy, David A. Brent, Nadine M. Melhem

**Affiliations:** 1 University of Pittsburgh Medical Center, USA; 2 University of Pittsburgh Graduate School of Public Health, USA; 3 University of Pittsburgh School of Medicine, USA; 4 University of Pittsburgh, USA; 5 Western Psychiatric Institute and Clinic of UPMC, USA

**Keywords:** suicide, psychiatric patients, neurocognition, cognition, implicit association, motor speed, response inhibition, executive function

## Abstract

**Background:**

Predicting suicide risk remains a challenge. We examined whether neurocognitive performance on implicit associations toward suicide, motor speed, response inhibition, and executive functioning predicts suicide attempt and behavior in high-risk psychiatric patients.

**Method:**

Our sample (*N* = 298) consisted of inpatients (*n* = 161) and outpatients (*n* = 83) admitted for a suicide attempt (SA; *n* = 78), for suicidal ideation (SI; *n* = 76), or were non-suicidal psychiatric controls (PC; *n* = 90), and healthy controls (HC; *n* = 54). Participants were followed for 12 months, with follow-up assessments at 3-, 6-, and 12-months. Neurocognitive tasks were administered at baseline. Clinical symptom measures, suicidality, and electronic health record data were collected at each timepoint. ANCOVA was used to compare groups on neurocognitive performance, and logistic and Cox regressions examined whether neurocognitive performance predicted future actual suicide attempt and suicidal behaviors.

**Results:**

Participants had a mean age of 24.34 years (*SD* = 3.71). A total of 19 participants made an actual suicide attempt during the study. On neurocognitive tasks at baseline, the SA group had stronger implicit associations with death- and suicide-related words compared to the HC (*d* = 0.88, *p* < 0.001) and SI (*d* = 0.63, *p* = 0.005) groups and poorer executive functioning than the SI (*d* = 0.44, *p* = 0.043) group in multivariate models. Stronger implicit associations with death/suicide predicted higher risk of suicide attempts at the univariate (*HR =* 1.68 *p* = 000), but not multivariate level (*HR =* 1.17 *p* = 000), while slower motor speed predicted actual suicide attempts (*HR =* 1.81 *p* = 000) at the multivariate level.

**Conclusions:**

Slower motor speed predicts actual suicide attempt and may help identify psychiatric patients who are at high risk for suicidal behavior.

## Introduction

Suicide is one of the leading causes of death worldwide in the United States (U.S.; CDC, 2021; World Health Organization, 2021). The risk of suicide is especially high in the year following psychiatric hospitalization (Chung et al., 2017; Haglund et al., 2019; O’Connell, Durns, & Kious, 2021). Predicting suicide, however, remains a challenge (Chung et al., 2017; Glenn & Nock, 2014). Risk assessment often relies on self-report, but the majority of patients who go on to die by suicide deny suicidal intent to healthcare providers prior to their death (Berman, 2018; Busch, Fawcett, & Jacobs, 2003). Thus, identifying *objective* markers of risk may improve our ability to predict suicidal behaviors. Neurocognitive tasks measuring implicit cognitions related to suicide, executive function, and inhibitory control can differentiate those with a history of suicidal behaviors from those without and predict future suicidal behaviors (Keilp et al., 2013; Moreno, Gutiérrez-Rojas, & Porras-Segovia, 2022; Saffer & Klonsky, 2018; Sörberg et al., 2013). Whether neurocognitive performance will improve our ability to predict suicidal behavior above and beyond established clinical risk factors such as anhedonia, childhood trauma, and social support is unclear (Auerbach, Pagliaccio, & Kirshenbaum, 2022; Kleiman & Liu, 2013; McLaughlin, Conron, Koenen, & Gilman, 2010; O’Connor, Gartland, & O’Connor, 2020).

The Death/Suicide Implicit Association Test (D/S-IAT) is one of the most commonly used implicit tasks used in suicide research (Moreno et al., 2022). A meta-analysis found that stronger associations toward death/suicide-related stimuli on the D/S-IAT scores differentiated individuals with and without a history of attempt (Sohn, McMorris, Bray, & McGirr, 2021). Bias toward death/suicide prospectively predicted future attempts, with a six-fold increase in the odds of attempt up to 6 months later, in inpatient and outpatient samples (Nock et al., 2010). Another popular implicit task is the Suicide Stroop (Nock et al., 2010). Increased bias toward suicide/death-related and words prospectively predicted future attempts up to 6 months in clinical and non-clinical adult samples (Cha, Najmi, Park, Finn, & Nock, 2010; Chung & Jeglic, 2017), although subsequent studies found otherwise (Mandel, Mitchell, Krush, & Revzina, 2025).

Studies examining executive functioning have been mixed. Lower executive functioning has been associated with attempt and its lethality (Keilp et al., 2013). Deficits in spatial planning ability, working memory, and motor functioning on simple motor speed tasks also predict suicidal ideation and/or behaviors (Keilp et al., 2013; Pu, Setoyama, & Noda, 2017; Riera-Serra et al., 2023), For example, slower motor speed has been shown to predict suicidal behaviors in psychiatric inpatients and outpatients (Canal-Rivero et al., 2018) suggesting that it might be a marker of suicide risk; although, other evidence suggests that slower motor speed may be specific to depression, not suicidal behaviors (Keilp et al., 2013). Finally, some findings suggest that greater response inhibition on the Go/No-Go task is associated with prior history of suicidal behaviors (Myers et al., 2023), although findings are mixed (MacPherson et al., 2022).

In this study, we examine the utility of neurocognitive tasks to predict suicidal behaviors in a sample of high-risk young adults enriched with psychiatric inpatients. Using a battery of neurocognitive tasks, we hypothesized that psychiatric patients admitted for suicide attempt would have stronger implicit cognitions toward suicide, poorer executive functioning, and reduced response inhibition, compared to psychiatric patients without suicidal ideation and behaviors and healthy controls. We also hypothesized that poorer neurocognitive functioning would predict suicidal behaviors over the year post-discharge.

## Method

### Sample

Participants in the current study were from a larger R01 study that aimed to identify objective predictors of suicidal behaviors, including biological measures of stress and immune functioning, and neurocognitive measures. Given the aims of the broader study, exclusion criteria included conditions that could affect the immune system. The sample consisted of psychiatric inpatients, outpatients, and healthy controls. Psychiatric inpatients were recruited from the University of Pittsburgh Medical Center (UPMC)’s Western Psychiatric Hospital across the spectrum of psychopathology and who were admitted for a suicide attempt (SA), admitted for suicidal ideation (SI), and psychiatric controls admitted without current suicidal ideation or behavior (PC). SI, PC, and healthy controls (HC) were also recruited through a university participant registry. SI and PC participants were included regardless of past suicidal behaviors, while the HC group had no history of psychiatric disorders and suicidal ideation and behaviors. Participants were assessed at intake/baseline, 3-, 6-, and 12-months. This study was reviewed and approved by the University of Pittsburgh Institutional Review Board.

A total of 515 participants aged 18–30 years were recruited into the study, of which 399 completed their baseline assessment. Of these, 298 participants completed at least one neurocognitive assessment at baseline and were included in the analyses. The final sample (*N* = 298) consisted of 78 SA, 76 SI, 90 PC, and 54 HC. Compared to those who had neurocognitive data at baseline, those who did not had a lower proportion of HC participants (*d =* 0.25), a higher proportion of SI participants (*d =* 0.31), higher levels of anxiety symptoms (*d =* 0.25), apathy (*d =* 0.30), and impulsivity (*d* = 0.39) (Supplementary Table S1).

Overall, 21% (*n* = 69) of the sample completed the study (i.e. completed baseline and all follow-up assessments). Of the remaining participants, 18% (*n* = 53) withdrew from the study and 59% (*n* = 176) were lost to follow-up. Supplementary Table S2 shows retention rates by group. We compared participants who only completed one assessment to those who completed more than one assessment and found the former more likely to be in the SA group (*d* = 0.56), less likely to be in the HC group (*d* = 0.47), had lower SES (*d* = 0.74), and higher rates of mood disorders (*d* = 0.43), anxiety disorders (*d* = 0.36), and substance use disorders (SUDs; *d* = 0.50). Those lost to follow-up also had more severe psychiatric symptoms (Supplementary Table S3).

### Demographic and clinical measures

Participants completed a standardized clinical interview during intake using the Structured Clinical Interview for DSM-5 (SCID). Diagnoses were confirmed following consensus meetings between the clinical interviewer and a licensed psychiatrist and supplemented by diagnoses from the electronic health records (EHR). Socioeconomic status (SES) was measured using the Hollingshead scale, a composite score based on years of education and income level, with higher numbers indicating higher levels of SES (Hollingshead, 1975). The Columbia Suicide Severity Rating Scale (C-SSRS) was used to assess suicidal thoughts and behaviors (Posner et al., 2011). We also reviewed the EHR for suicidal behaviors.

Clinical symptoms were assessed using measures that are often associated with suicide risk: the Patient Health Questionnaire (Spitzer, Kroenke, Williams, & Group, 1999), Generalized Anxiety Disorder-7 scale (Spitzer, Kroenke, Williams, & Löwe, 2006), the Post-traumatic Stress Disorder (PTSD) Checklist for the Diagnostic and Statistical Manual of Mental Disorders—5^th^ Edition (Weathers et al., 2013), the Beck Hopelessness Scale (Beck, Weissman, Lester, & Trexler, 1974), the Patient Health Questionnaire-15 (Spitzer et al., 1999), the Apathy Evaluation Scale (Marin, Biedrzycki, & Firinciogullari, 1991), the Buss-Perry Aggression Questionnaire (Buss & Perry, 1992), the Barratt Impulsivity Scale (Patton, Standord, & Barratt, 1995), the Affective Lability Scale (Harvey, Greenberg, & Serper, 1989), the Perceived Stress Scale (Cohen, Kamarck, & Mermelstein, 1983), the Snaith-Hamilton Pleasure Scale (Snaith et al., 1995), the Drug Use Screening Inventory (Tarter & Kirisci, 1997), and the insomnia rating scale from the PhenX Toolkit (Hamilton et al., 2011).

### Risk and protective factors

Risk and protective factors for suicide were assessed using the Childhood Trauma Questionnaire (CTQ) (Bernstein, Fink, Handelsman, & Foote, 2011), and the Multidimensional Scale of Perceived Social Support (Zimet, Dahlem, Zimet, & Farley, 1988) as these factors are related to suicide risk (Kleiman & Liu, 2013; Zatti et al., 2017).

### Neurocognitive tasks

Participants completed a battery of computer-based neurocognitive tasks at baseline, assessing implicit biases toward suicide, executive functioning, and response inhibition. The Wechsler Abbreviated Scale of Intelligence 2nd Edition (WASI-II)(Wechsler, 2018) was used as a measure of intelligence quotient (IQ), which was used as a covariate in analyses predicting neurocognitive performance.

*Suicide-related tasks.* The following tasks were used to assess implicit cognitive and attentional biases toward death and suicide, which prior studies suggest is impaired in suicidal populations and predict suicidal behaviors (Barnes et al., 2017; Chung & Jeglic, 2017; Nock et al., 2010).

*Death/Suicide Implicit Associations Test (D/S IAT).* Suicide-related cognitions were assessed via the D/S IAT (Nock et al., 2010), which measures an individual’s implicit thoughts about life and death/suicide. Participants are presented with varying combinations of the constructs ‘life’ and ‘death/suicide’ as well as the attributes ‘me’ and ‘not me’. The D/S IAT provides *D* scores representing the association between ‘death’ and ‘me’, with more positive scores indicating a stronger implicit association. *D* scores were calculated by taking the difference between mean reaction times for death/suicide words and me/not me words, divided by the pooled standard deviation.

*Suicide Stroop Task.* Attentional bias toward suicide was assessed via a modified Suicide Stroop task (Cha et al., 2010), which asks subjects to determine the color of words as quickly and as accurately as possible. Interference scores for suicide/death-related were calculated by comparing reaction times for suicide-related versus neutral words.

*CANTAB Assessments.* The Cambridge Neuropsychological Test Automated Battery (CANTAB) (Sahakian & Owen, 1992) includes tasks that assess executive functioning, working memory, and response inhibition, which have been previously reported to be impaired in suicidal populations (Fernández-Sevillano et al., 2021; Riera-Serra et al., 2023) and, for executive functioning and working memory, predictive of suicidal ideation and behaviors (Keilp et al., 2013; Pu et al., 2017; Riera-Serra et al., 2023). For all CANTAB tasks, scores were normalized by converting raw scores to percentiles because participants completed the tasks either through the CANTAB website or the iPad app and the distribution of scores differed between methods.

*Motor Screening Task (MOT).* The MOT is administered first to assess for sensorimotor and cognitive deficits that could impede further testing (Cambridge Cognition Ltd, 2023a). Participants are presented with colored crosses on a black screen and are asked to touch the crosses with the forefinger of their dominant hand as the crosses appear. The outcome measure for the MOT task was the mean latency of trial responses (MOT mean latency), that is, motor speed.

*One Touch Stockings of Cambridge (OTS).* The OTS task (Cambridge Cognition Ltd, 2023b; Owen et al., 1990) gives participants two sets of colored balls in the upper and lower portions of the screen that appear to be hanging by strings attached to two beams with numbered boxes underneath. Participants move the balls in the lower half of the screen so they match the balls at the top of the screen. The mean latency to correct answers (i.e. the length of time the participant took to reach the correct answer) was the primary outcome used in this study (OTS mean latency to correct).

*Spatial Working Memory (SWM) Task.* The SWM measures visuospatial functioning and working memory (Cambridge Cognition Ltd, 2023c; Corsi & Michael, 1972; Owen et al., 1990). Participants are presented with up to 12 colored boxes, one of which contains a yellow token that is not visible until the box is selected. Participants select each box and use the process of elimination to determine which box contains the yellow token. The color and position in which the boxes appear change with each trial. The primary outcome of the SWM task is the number of times a participant selects a box they have already selected within a trial while searching for the yellow token (SWM between errors).

*Stop Signal Task (SST).* Participants are presented with an arrow pointing left or right and are asked to respond by selecting the direction of the arrow as quickly as possible (Cambridge Cognition Ltd, 2023d; Lappin & Eriksen, 1966). For stop trials (50%), a sound plays as the arrow appears on the screen and the participant is instructed to withhold their response. Stop signal reaction time (SSRT) was our primary outcome, which is an estimate of the time it takes to inhibit a response. SSRT is calculated by subtracting the stop-signal delay (SSD) from the go reaction time corresponding to the proportion of stop trials on which participants failed to inhibit their response.

### Statistical analysis

All analyses were conducted in R version 4.5.0 (R Core Team, 2025). Missing data were not missing completely at random (MCAR; Little’s MCAR test: χ^2^(1269) = 1527.3, *p* < .001). However, those lost to follow-up, and as such had the highest degree of missing data, had more severe psychiatric problems (Supplementary Table S3). We used full information maximum likelihood estimation and multivariate imputation by chained equations from the *mice* package (van Buuren & Groothuis-Oudshoorn, 2011) to impute missing data with predictive mean matching (*m* = 5) and pooled effects according to Rubin’s rules (Royston, 2004). We conducted sensitivity analyses for our final models using complete data and compared results to those from the imputed datasets as evidence of the missingness at random (MAR) assumption for *mice.*

We compared SA, SI, PC, and HC participants on demographic characteristics and clinical and neurocognitive measures at baseline using Analysis of Variance (ANOVA), χ^2^, or Fisher’s exact tests. To correct for multiple comparisons, we used a Bonferroni-adjusted α level calculated based on the number of neurocognitive tasks analyzed (*n* = 6, *p* = 0.05/6 = 0.0083). We used Tukey-adjusted post-hoc comparisons for all group contrasts when group comparisons were significant at the Bonferroni-corrected level.

Due to the large number of intercorrelated clinical symptom measures, we used factor analysis with varimax rotation to reduce the data. Bartlett’s test of sphericity and the Kaiser–Meyer–Olkin tests were used to assess whether data were suitable for a factor analysis. Exploratory analysis (EFA) was first used to identify clinical factors and confirmatory factor analyses (CFAs) were conducted at each follow-up timepoint using the factor structure from the EFA. Measurement invariance was used to examine whether the factors were equivalent across timepoints.

For neurocognitive tasks, we used ANCOVA to examine whether there were group differences, controlling for covariates. We first assessed the relationship of each neurocognitive task to these covariates, including sociodemographic characteristics, clinical factor scores, and risk and protective factors. For analyses predicting neurocognitive performance at baseline, covariates significant at the *p* < .0083 level were added to the model in addition to controlling for IQ. Models were reduced in a stepwise fashion to achieve the most parsimonious model.

Finally, we examined whether neurocognitive performance at baseline prospectively predicted future suicidal behavior (*n* = 264). HC participants were excluded since none had any suicidal behaviors during the study. We used narrow and broad definitions of ‘actual suicide attempt’ and ‘suicidal behavior’, where the latter included actual, interrupted, aborted attempts, preparatory behaviors, and emergency referrals. Logistic regression was used to prospectively predict actual suicide attempts and suicidal behaviors using baseline measures. Cox regression was used to predict time-to-onset for each of the actual suicide attempts and suicidal behavior using time-varying covariates. For analyses predicting actual suicide attempts and suicidal behaviors, covariates associated with the outcome were added to models and then reduced before adding the neurocognitive task variable to examine whether it was predictive above and beyond covariates. For logistic and Cox models, we determined significance at α-level = 0.05.

## Results

### Baseline group comparisons and data reduction

Table 1 presents sample characteristics and group comparisons (SA/SI/PC/HC). Groups differed in terms of SES, with the SI and SA groups having lower SES than the HC and PC groups. In general, groups differed such that HC < PC < SA = SI in terms of higher rates of psychiatric diagnoses, higher levels of clinical symptoms, higher levels of risk factors, and lower levels of protective factors. The SI and SA groups were similar on all measures except that the SA group had lower IQ scores than the SI group and had a higher proportion of actual suicide attempt during the study (18% versus 5%).

**Table 1.** Sample sociodemographic, psychiatric, and clinical characteristics with suicide outcomes and group comparisons

Characteristic	*N*	*M*(SD) or *n*(%)					Test	Statistic	*p*
		Overall	SA	SI	PC	HC			
		*N* = 298	*n* = 78	*n* = 76	*n* = 90	*n* = 54			
Sociodemographic characteristics									
Age	298	24.34(3.71)	23.63(3.88)	24.61(4.06)	24.57(3.32)	24.61(3.53)	*F*(3,294)	1.20	0.312
Sex (male)	298	116(39%)	31(40%)	31(41%)	30(33%)	24(44%)	χ^2^(3)	2.01	0.571
Race (White versus Non-White)^a^	297	213(72%)	48(62%)	60(79%)	63(70%)	42(78%)	χ^2^(3)	6.41	0.093
American Indian	297	1(0.3%)	1(0.3%)	0(0%)	0(0%)	0(0%)	Fisher’s	–	0.697
Asian	297	23(7.7%)	5(6.5%)	6(7.9%)	6(6.7%)	6(11%)	Fisher’s	–	0.736
Black	297	38(13%)	17(22%)*^a^*	5(6.6%)*^b^*	13(14%)*^ab^*	3(5.6%)*^b^*	χ^2^(3)	11.30	0.010
White	297	213(72%)	48(62%)	60(79%)	63(70%)	42(78%)	χ^2^(3)	6.41	0.093
Multiracial	297	22(7.4%)	6(7.8%)	5(6.6%)	8(8.9%)	3(5.6%)	Fisher’s	–	0.908
Ethnicity (Hispanic)	297	26(8.8%)	7(9.1%)	11(14%)	7(7.8%)	1(1.9%)	Fisher’s	–	0.082
SES	283	−0.16(0.98)	−0.64(0.94)*^b^*	−0.38(0.91)*^b^*	0.09(0.88)*^a^*	0.39(0.90)*^a^*	*F*(3,279)	16.51	<0.001*
LGBTQ	273	91(33%)	28(40%)*^a^*	29(43%)*^a^*	28(34%)*^a^*	6(11%)*^b^*	χ^2^(3)	15.64	0.001*
IQ (WASI-II)	247	112.63(16.11)	104.02(15.35)*^b^*	114.65(15.28)*^a^*	112.91(16.30)*^a^*	119.06(13.96)*^a^*	*F*(3,243)	9.80	<0.001*
Psychiatric characteristics									
Mood disorder	281	209(74%)	73(95%)*^a^*	66(92%)*^a^*	70(86%)*^a^*	0(0%)*^b^*	χ^2^(3)	182.36	<0.001*
Anxiety disorder	281	126(45%)	28(36%)*^a^*	39(54%)*^a^*	59(73%)*^b^*	0(0%)*^c^*	χ^2^(3)	71.90	<0.001*
PTSD	281	64(23%)	25(32%)*^a^*	20(28%)*^a^*	19(23%)*^a^*	0(0%)*^b^*	χ^2^(3)	20.20	<0.001*
Psychosis	281	9(3.2%)	2(2.6%)	5(6.9%)	2(2.5%)	0(0%)	Fisher’s	–	0.227
SUD	281	136(48%)	44(57%)*^a^*	49(68%)*^a^*	42(52%)*^a^*	1(2.0%)*^b^*	χ^2^(3)	57.92	<0.001*
Clinical measures									
Affective liability (ALS)	293	1.24(0.84)	1.77(0.65)*^a^*	1.60(0.70)*^a^*	1.11(0.74)*^b^*	0.20(0.23)*^c^*	*F*(3,289)	198.95	<0.001*
Apathy	274	39.33(7.87)	41.62(7.11)*^a^*	43.93(7.85)*^a^*	38.73(6.61)*^b^*	31.53(3.59)*^c^*	*F*(3,270)	68.30	<0.001*
Hopelessness (BHS)	296	9.66(2.33)	10.57(2.45)*^a^*	10.04(2.03)*^ab^*	9.32(2.39)*^bc^*	8.42(1.73)*^c^*	*F*(3,292)	13.79	<0.001*
Impulsivity (BIS)	269	63.65(13.86)	71.21(12.28)*^a^*	70.31(13.51)*^a^*	60.68(11.20)*^b^*	50.66(7.61)*^c^*	*F*(3,265)	57.12	<0.001*
Aggression (BPS)	293	0.47(0.16)	0.55(0.16)*^a^*	0.53(0.14)*^a^*	0.45(0.13)*^b^*	0.31(0.07)*^c^*	*F*(3,289)	71.82	<0.001*
Anxiety (GAD–7)	296	8.92(6.55)	12.55(5.13)*^a^*	12.32(5.64)*^a^*	7.90(5.41)*^b^*	0.72(1.35)*^c^*	*F*(3,292)	230.65	<0.001*
Insomnia	274	10.72(7.41)	15.07(6.04)*^a^*	13.31(6.72)*^a^*	10.05(6.95)*^b^*	3.00(3.21)*^c^*	*F*(3,270)	89.25	<0.001*
Depression (PHQ–9)	296	11.24(8.44)	16.46(6.53)*^a^*	17.15(6.40)*^a^*	8.16(5.94)*^b^*	0.73(1.08)*^c^*	*F*(3,292)	319.12	<0.001*
Somatic symptoms (PHQ–15)	296	7.66(5.23)	9.89(4.39)*^a^*	10.09(4.98)*^a^*	7.11(4.63)*^b^*	2.02(2.29)*^c^*	*F*(3,292)	91.18	<0.001*
Perceived stress (PSS)	270	21.45(9.17)	27.55(5.29)*^a^*	26.83(6.69)*^a^*	20.13(6.80)*^b^*	8.96(5.08)*^c^*	*F*(3,266)	146.64	<0.001*
PTS Symptoms (PCL)	282	28.59(22.75)	44.01(17.46)*^a^*	38.08(20.60)*^a^*	23.32(18.78)*^b^*	1.56(2.39)*^c^*	*F*(3,278)	239.08	<0.001*
Anhedonia (SHAPS)	272	2.84(2.96)	4.00(3.23)*^a^*	4.22(2.99)*^a^*	1.99(2.36)*^c^*	1.00(1.80)*^c^*	*F*(3,268)	24.97	<0.001*
Substance use (DUSI-a)	292	21.35(4.98)	23.25(5.62)*^a^*	22.34(5.37)*^ab^*	20.91(4.42)*^b^*	18.07(1.41)*^c^*	*F*(3,288)	36.74	<0.001*
Substance use problems (DUSI-b)	291	25.41(30.51)	41.08(34.90)*^a^*	33.06(32.57)*^a^*	18.50(23.61)*^b^*	4.81(9.64)*^c^*	*F*(3,287)	39.34	<0.001*
Clinical factors									
Factor 1	248	−0.02(1.13)	0.58(0.99)*^a^*	0.45(1.03)*^a^*	−0.12(1.08)*^b^*	−1.15(0.34)*^c^*	*F*(3,244)	93.43	<0.001*
Factor 2	248	−0.02(1.16)	0.28(1.28)*^a^*	0.70(1.15)*^a^*	−0.31(0.93)*^b^*	−0.84(0.52)*^c^*	*F*(3,244)	36.03	<0.001*
Factor 3	248	−0.09(1.04)	0.31(1.14)*^a^*	0.08(0.91)*^a^*	−0.10(1.10)*^a^*	−0.74(0.60)*^b^*	*F*(3,244)	18.15	<0.001*
Factor 4	248	−0.04(1.25)	0.40(1.55)*^a^*	0.17(1.43)*^ab^*	−0.28(1.08)*^b^*	−0.42(0.48)*^b^*	*F*(3,244)	6.76	<0.001*
Risk and protective factors									
Childhood Trauma (CTQ)	291	57.83(10.60)	61.51(12.73)*^a^*	60.49(12.34)*^a^*	55.89(7.55)*^b^*	51.98(3.35)*^b^*	*F*(3,287)	23.77	<0.001*
Social Support (MSPSS)	273	5.38(1.26)	4.85(1.37)*^c^*	4.93(1.14)*^c^*	5.45(1.10)*^b^*	6.49(0.63)*^a^*	*F*(3,269)	47.44	<0.001*
Suicide outcomes at 12 months									
Actual suicide attempt	298	19(6.4%)	14(18%)*^a^*	4(5.3%)*^b^*	1(1.1%)*^b^*	0(0%)*^b^*	Fisher’s	–	<0.001*
Suicidal behaviors	298	43(14%)	22(28%)*^a^*	16(21%)*^a^*	5(5.6%)*^b^*	0(0%)*^b^*	χ^2^(3)	29.53	<0.001*

*Note:* *p < 0.05. Frequencies are observed frequencies. ^a^Given the small number of non-White participants, we separated participants as White and non-White for analyses. Superscript letters in italic format denote significant post-hoc group differences, where differing letters between groups (e.g. a versus b or ab versus c) indicate a significant (*p* < 0.05) difference. SA, suicide attempt group; SI, suicidal ideation group; PC, psychiatric control group; HC, healthy control group. SES, Hollingshed scale; WASI-II, Weschler Abbreviated Scale of Intelligence, 2nd edition; PTSD, Posttraumatic Stress Disorder; SUD, Substance Use Disorder; ALS, Affective Liability Scale; Apathy, Apathy Evaluation Scale; BHS, Beck Hopelessness Scale; BIS, Barratt Impulsivity Scale; BPS, Buss–Perry Aggression Questionnaire; GAD-7, Generalized Anxiety Disorder scale; Insomnia, Insomnia PhenX Toolkit; PHQ-9, Patient Health Questionnaire; PHQ-15, Patient Health Questionnaire; PSS, Perceived Stress Scale; PCL, PTSD Checklist for the DSM-5; SHAPS, Snaith-Hamilton Pleasure Scale; DUSI, Drug Use Screening Inventory; CTQ, Childhood Trauma Questionnaire; MSPSS, Multidimensional Scale of Perceived Social Support.

Correlations between the clinical measures can be found in Supplementary Table S4. Bartlett’s test of sphericity (*p* < 0.001) and the Kaiser-Meyer-Olkin (KMO) test (KMO = 0.93) indicated that the data were appropriate for factor analyses. Parallel analyses indicated that a four-factor solution fit the data best. Factors generally represented 1) psychological distress, 2) apathy and anhedonia, 3) emotion regulation, and 4) substance use. Factor loadings can be found in Supplementary Table S5. To confirm that the inclusion of missing data when estimating the covariance matrix did not affect the EFA, factor loadings for analyses with and without missing data were compared (Supplementary Table S5). Using the same factor model identified in the EFA, factor scores for follow-up timepoints were extracted using confirmatory factor analyses (CFA). While fit measures did not pass all established thresholds, the factor structure was retained given each factor’s clinical significance (Supplementary Tables S6 and S7). Results indicated evidence for scalar invariance, that is, the factor loadings and factor intercepts did not appear to change over time.

### Group comparisons on baseline neurocognitive performance

There were significant group differences on the D/S-IAT, MOT Mean Latency, and OTS Latency to Correct at *p* < 0.0083 (Table 2). There were no significant group differences for the Suicide Stroop, SWM Between Errors, and SSRT on the SST.

**Table 2.** Group differences for neurocognitive tasks

	*N*	*M*(SD)				Test	Value	*p*
		SA (*n* = 78)	SI (*n* = 76)	PC (*n* = 90)	HC (*n* = 54)			
D/S-IAT	251	−0.10(0.38)^a^	−0.27(0.41)^ab^	−0.29(0.42)^b^	−0.48(0.34)^c^	*F*(3, 247)	8.32	<0.001*
Stroop suicide interference	245	−5.67(118.42)	−20.94(230.78)	24.16(95.74)	15.05(79.80)	*F*(3, 241)	1.27	0.286
MOT mean latency (pct)	283	0.57(0.29)^a^	0.57(0.31)^a^	0.49(0.31)^ab^	0.40(0.26)^b^	*F*(3, 279)	5.08	0.002*
OTS mean latency to correct (pct)	276	0.48(0.29)^a^	0.63(0.28)^b^	0.57(0.27)^ab^	0.49(0.26)^a^	*F*(3, 272)	4.37	0.005*
SST SSRT (pct)	280	0.58(0.30)	0.57(0.27)	0.49(0.29)	0.51(0.29)	*F*(3, 276)	1.66	0.177
SWM between errors (pct)	283	0.60(0.26)	0.62(0.27)	0.56(0.27)	0.47(0.30)	*F*(3, 279)	3.58	0.014

*Note:* *p < 0.0083. Tukey-adjusted differences between groups. Superscript letters denote significant post-hoc group differences, where differing letters between groups (e.g. a versus b or ab versus c) indicates a significant (*p* < 0.05) difference. Tasks marked with pct were converted to percentile scores. SA, suicide attempt group; SI, suicidal ideation group; PC, psychiatric control group; HC, healthy control group. D/S IAT, Death/Suicide Implicit Association Test; MOT, Motor Screening Task; OTS, one touch stocking task; SST, stop signal task; SSRT, stop signal reaction time; SWM, spatial working memory task.

#### Death/Suicide Implicit Association Test (D/S-IAT)

At the *p* < .0083 level, groups significantly differed on the D/S-IAT (Cohen’s *f* = 0.32, *p* < 0.001; Table 2). Post-hoc comparisons (Supplementary Table S8) showed that the SA group had significantly higher D/S-IAT scores than both the PC (*d* = 0.47, *p* = 0.034) and HC groups (*d* = 0.95, *p* < .001), with higher scores indicating a stronger association with suicide- and death-related words. The SI group also had higher scores than the HC group (*d* = 0.54, *p* = 0.023), as did the PC group (*d* = 0.48, *p* = 0.040). The SA and SI groups did not differ from each other, nor did the SI and PC groups.

Next, we identified the sociodemographic and clinical factors that were associated with D/S-IAT scores at baseline, pooling across imputed datasets (Supplementary Table S9). Clinical higher levels of psychological distress (Factor 1; *β* = 0.17, *SE* = 0.06, *p* = 0.003), higher levels of apathy and anhedonia (Factor 2; *β* = 0.26, *SE* = 0.06, *p* < 0.001), Mood disorders (*β* = 0.56, *SE* = 3.93, *p* < 0.001), and lower levels of social support (*β* = −0.22, *SE =* 0.06, *p* = 0.001) predicted higher D/S-IAT scores at baseline. Only Factor 2 remained in the final model (Table 3), and the effect of group remained significant (*f* = 0.08, *p* < 0.001). Higher levels of apathy and anhedonia also predicted higher *D* score on the D/S-IAT. Post-hoc comparisons found that the SA group had a stronger association with death and suicide-related words than the HC (*d* = 0.88, *p* < 0.001), PC (*d* = 0.47, *p* = 0.048), and SI (*d* = 0.63, *p* = 0.005) groups even after controlling for covariates.

**Table 3.** Pooled and reduced ANCOVA models examining the differences in performance on neurocognitive tasks at baseline by group

Parameter	*SS*	*F*	*Df*	β*(SE)*	*p*	Cohen’s *f*
Model: D/S-IAT: *R*^2^ = 0.16, *F*(5, 173.71) = 8.72, *p* = < 0.001						
Group	17.55	6.49	3		< 0.001***	0.08
IQ (WASI-II)	4.10	3.77	1	0.13(0.07)	0.053	0.02
Factor 2	10.81	12.06	1	0.21(0.06)	< 0.001***	0.05
Residuals	215.56		173.71			
Model: MOT mean latency (pct): *R*^2^ = 0.10, *F*(4, 229.02) = 7.89, *p* = < 0.001						
Group	9.03	3.26	3		0.021*	0.04
IQ (WASI-II)	14.94	14.97	1	−0.24(0.06)	< 0.001***	0.06
Residuals	255.58		229.02			
Model: OTS mean latency to correct (pct): *R*^2^ = 0.11, *F*(6, 207.05) = 5.08, *p* < 0.001						
Group	9.45	3.34	3		0.018*	0.04
IQ (WASI-II)	1.88	1.58	1	0.08(0.06)	0.209	0.01
Age	8.05	8.54	1	0.18(0.06)	0.003**	0.03
PTSD	4.60	4.69	1	0.33(0.15)	0.030*	0.02
Residuals	252.89		207.05			

*Note:* *p < 0.05, **p < 0.01, ***p < 0.001. Effects are standardized and pooled. Tasks marked with pct were converted to percentile scores. Cohen’s f, Partial Cohen’s f; D/S-IAT, Death/Suicide Implicit Association Test; MOT, Motor Screening Task; OTS, One Touch Stocking task; WASI-II, Weschler Abbreviated Scale of Intelligence; MSPSS, Multidimensional Scale of Perceived Social Support; PTSD, posttraumatic stress disorder.

#### Motor Screening Task (MOT) Mean Latency

Groups significantly differed on the MOT task’s mean latency score at baseline (*f* = 0.23, *p* = 0.002,) at the *p* < .0083 level (Table 2). The SA (*d* = 0.61, *p* = .004) and SI (*d* = 0.61, *p* = 0.005) groups had significantly longer response latencies than the HC group, while the remaining group contrasts were non-significant (Supplementary Table S8).

Of the sociodemographic and clinical covariates, lower SES (*β* = −0.19, *SE* = 0.06, *p =* 0.003), higher levels of Factor 1 (i.e. generally psychological distress; *β* = 0.18, *SE* = 0.05, *p =* 0.001), Mood disorders (*β =* 0.39, *SE =* 0.14, *p =* 0.004), SUDs (*β =* 0.37, *SE =* 0.12, *p =* 0.002), and lower levels of social support (*β =* −0.17, *SE =* 0.06, *p =* 0.005) predicted longer latencies on the MOT (Supplementary Table S9). In the final model, the only significant covariate left in the model was IQ (*β* = −0.21, *SE* = 0.07, *p* = 0.002). None of the covariates survived after reducing the model and the effect of group remained significant (*f =* 0.04, *p* = 0.002) (Table 3). Post-hoc comparisons found that the HC and SI groups differed such that the SI group had longer latencies than the HC group (*d =* 0.54, *p =* 0.16).

#### One Touch Stocking Task (OTS) mean latency to correct

On the OTS task, groups differed in their OTS mean latency to correct scores (*f* = 0.22, *p* = 0.005). The SI group had significantly longer latencies to correct responses on the OTS than the SA (*d* = 0.53, *p* = 0.010) and HC (*d =* 0.50, *p =* 0.031) groups (Supplementary Table S8).

At the univariate level, older age (*β =* 0.20, *SE =* 0.06, *p =* 0.001) and PTSD (*β =* 0.40, *SE =* 0.15, *p =* 0.007) were associated with longer latencies to correct on the OTS (Supplementary Table S9). In the final reduced model, the effect of group remained significant (*f* = 0.04, *p* = 0.018). Older age (*β* = 0.18, *SE* = 0.06, *p* = 0.003), and current PTSD diagnoses (*β* = 0.33, *SE* = 0.15, *p* = 0.030) also predicted longer latencies on the OTS at baseline (Table 3). Between groups, the SA and SI groups significantly differed (*d* = 0.46, *p* = 0.043) such that the SI group had longer latencies than the SA group.

### Prediction of 12-month suicide outcomes

A total of 19 (7.8%) participants (excluding healthy controls) reported an actual suicide attempt during the study. The SA group was significantly more likely to have an actual attempt prospectively (*n* = 14, 18%) compared to SI (*n* = 5, 6.1%), and PC (*n* = 1, 1%). A total of 43(17.6%) participants reported suicidal behaviors during the study period. The SA (*n* = 22, 28%) and SI (*n* = 16, 21%) groups were significantly more likely to have suicidal behavior prospectively compared to PC (*n* = 5, 5.6%) (Table 1). Supplementary Table S10 presents comparisons between those with and without a prospective actual attempt, and similarly for prospective suicidal behaviors.

*Clinical model.* At the univariate level, time-varying cox models found that lower SES (*HR* = 0.47, *SE* = 0.14, *p* = 0.009), LGBTQ identities (*HR =* 2.97, *SE* = 1.80, *p =* 0.034), higher levels of Factor 2 (*HR =* 1.58, *SE* = 0.31, *p =* 0.017), current PTSD diagnoses (*HR =* 2.51, *SE* = 1.32, *p* = 0.046), and lower levels of social support (*HR =* 0.40, *SE* = 0.09, *p <* 0.001) increased the relative risk of making an actual suicide attempt during the study (Supplementary Table S11). In the final clinical model, only SES (*HR =* 0.54, *SE* = 0.15, *p =* 0.24) and social support (*HR =* 0.62, *SE* = 0.14, *p =* 0.033) predicted actual suicide attempts (AUC = 0.86, *SE* = 0.07). We next examined the performance on each of the neurocognitive tasks at baseline in predicting actual attempt when added to the clinical model.

*Actual suicide attempts.* In univariate Cox models, baseline D/S-IAT scores positively predicted risk of actual attempts over time (*HR =* 1.68, *SE* = 0.44, *p =* 0.041) and longer mean latencies on the MOT task also predicted higher risk of actual attempts over time (*HR =* 1.72, *SE* = 0.47, *p =* 0.038; Table 4). Univariate logistic regressions predicting prospective actual attempt and suicidal behavior as using neurocognitive performance at baseline found similar results (Supplementary Table S12).

**Table 4.** Cognitive tasks predicting time-to-onset for actual suicide attempts and suicidal behaviors—univariate cox models

Task	Actual suicide attempt			Suicidal behaviors		
	HR (SE)	95% CI	*p*	HR (SE)	95% CI	*p*
D/S-IAT	1.68(0.44)	1.02, 2.75	0.041*	1.20(0.20)	0.87, 1.66	0.268
Stroop: suicide interference	1.14(0.34)	0.65, 2.00	0.639	1.01(0.18)	0.71, 1.42	0.971
MOT mean latency (pct)	1.72(0.47)	1.03, 2.87	0.038*	1.29(0.21)	0.94, 1.76	0.113
OTS mean latency to correct (pct)	0.74(0.17)	0.47, 1.15	0.184	0.96(0.15)	0.71, 1.29	0.776
SST SSRT (pct)	1.35(0.34)	0.84, 2.17	0.217	1.27(0.21)	0.93, 1.74	0.132
SWM between errors (pct)	0.69(0.17)	0.42, 1.11	0.124	0.87(0.14)	0.63, 1.19	0.370

*Note:* *p < 0.05. HRs are standardized. Tasks marked with pct were converted to percentile scores. SA, actual D/S IAT, Death/Suicide Implicit Association Test; MOT, Motor Screening Task; OTS, One Touch Stocking task; SST, Stop Signal Task; SSRT, Stop Signal Reaction Time; SWM, Spatial Working Memory Task.

When the D/S-IAT was added to the clinical Cox model, D/S-IAT scores did not predict attempts (*HR =* 1.17, *SE* = 0.34, *p =* 0.557) (Table 5). In logistic models, compared to the AUC of the clinical model (AUC = 0.84, *SE* = 0.07), the addition of the D/S-IAT (AUC = 0.84, *SE* = 0.08) did not improve the model’s performance (*z* = −0.55, *p =* 0.593) (Supplementary Tables S13 and S14).

**Table 5.** Cognitive tasks predicting time-to-onset for actual suicide attempts—pooled multivariate cox models

D/S-IAT			
Parameter	HR(SE)	95% CI	*p*
D/S-IAT	1.17(0.34)	0.69, 2.01	0.557
SES	0.58(0.15)	0.36, 0.94	0.027*
Social support (MSPSS)	0.55(0.18)	0.30, 1.00	0.051

MOT mean latency			
Parameter	HR(SE)	95% CI	*p*
MOT mean latency (pct)	1.81(0.51)	1.07, 3.06	0.027*
SES	0.66(0.19)	0.38, 1.14	0.134
Social support (MSPSS)	0.57(0.14)	0.36, 0.90	0.016*

*Note:* *p < 0.05. Estimates are standardized and pooled. Tasks marked with pct were converted to percentile scores. D/S-IAT, Death/Suicide Implicit Association Test; MOT, Motor Screening Task; SES, Hollingshed Scale; MSPSS, Multidimensional Scale of Perceived Social Support.

When the MOT was added to the clinical Cox model, longer mean latencies longitudinally predicted greater risk of actual attempts (*HR =* 1.81, *SE* = 0.51, *p =* 0.027) (Table 5). Although, in logistic models, the addition of the MOT (AUC = 0.90, *SE* = 0.06) improved the AUC from 0.84 to 0.90, the difference between the models’ performance did not reach statistical significance (*z =* −1.70, *p =* 0.091) (Supplementary Tables S13 and S14).

*Suicidal behaviors.* None of the cognitive tasks were significantly associated or predicted suicidal behaviors (Table 4 and Supplementary Table S12).

### Sensitivity analyses

Supplementary Tables S15–S17 present results including participants with complete data. Similar findings were obtained. Since the MOT is a screening task, we also ran sensitivity analyses excluding participants with scores <1.15 SDs below the mean and found similar results (Supplementary Tables S18–S21).

## Discussion

At baseline, we found that those in the SA group had stronger implicit suicide-related associations compared to the HC and PC groups, even after controlling for sociodemographic and clinical covariates. The SA and SI groups were also significantly different on the OTS executive functioning task, whereby those in the SI group took longer to solve the task than the SA group, even after controlling for sociodemographic and clinical covariates. Prospectively, slower motor speed on the MOT was predictive of actual suicide attempts even after controlling for clinical predictors. None of the other neurocognitive tasks were predictive of suicidal behaviors.

The D/S-IAT has the strongest evidence for the ability to identify patients with a history of suicide attempt and predict future attempts or behaviors in young adults, adults, and Veterans (Barnes et al., 2017, *p.* 201; Ellis, Rufino, & Green, 2016; Glenn et al., 2017; Tello et al., 2020; Toukhy et al., 2025). Stronger implicit associations with death and suicide have been associated with six-fold higher odds of suicide attempt in the following 6 months (Nock et al., 2010). However, others have found that D/S-IAT did not predict suicide attempts in adolescents following a pediatric emergency room visit (Brent et al., 2023). Additionally, performance on the D/S-IAT was not associated with suicidal thoughts and behaviors among inpatients both at the time of admission and 6 months post-discharge, nor did performance on the D/S-IAT change between admission and the 6 month follow-up (Levy et al., 2025). While meta-analysis found that higher D/S-IAT scores were associated with a history of making a suicide attempt across adolescents, young adults, and adults, the effect was weaker among acute care patients (Sohn et al., 2021). We found stronger implicit suicide-related associations among attempters at the time of admission; however, it did not predict future attempts, corroborating prior findings of studies suggesting that the D/S-IAT may not be a useful indicator of suicide risk in psychiatric populations.

We found that slower motor speed was predictive of future suicide attempts. Our results are specific to actual attempts, whereas prior studies have shown that poorer motor dexterity, indicated by slower motor speed, is predictive of suicidal behaviors (Canal-Rivero et al., 2018). Other studies have also shown that slower motor speed was specific to depression and not suicidal behaviors (Keilp et al., 2013), and to predict suicidal ideation (Pu et al., 2017). We found that slower motor speed may be a useful objective marker of suicide risk. We also found that motor speed predicted actual attempts, but not suicidal behaviors, suggesting that slower motor speed may represent a higher severity of psychiatric presentation of actual attempters.

The SI group had poorer working memory and planning abilities, although neither the OTS nor the SWM predicted future attempts. While some have reported impaired spatial planning and working memory (Riera-Serra et al., 2023; Ruch et al., 2020). Others have found *better* executive functioning among those with a history of attempt compared to controls (Burton, Vella, Weller, & Twamley, 2011; Nangle et al., 2006). Furthermore, in adults, recent attempts were associated with better problem-solving ability but poorer inhibitory control (Burton et al., 2011), so the relationship between executive functioning and suicidal behavior may be more specific to certain domains of executive functioning.

### Strengths and limitations

A major strength of the study was its longitudinal design in a high-risk sample of psychiatric patients and the use of a range of neurocognitive assessments. One limitation was the relatively small number of participants who had an actual attempt prospectively (*n* = 19), which limited power to detect smaller effect sizes. Our retention rate was also low among this high-risk population, consistent with prior studies (Fischer, Dornelas, & Goethe, 2001; Gibbons, Stirman, Brown, & Beck, 2010; Klimas et al., 2021) and the severity of their psychiatric presentation. This population is notorious for the low retention even in the context of treatment where 44–64% of psychiatric inpatients miss their outpatient follow-up appointments post-discharge (Benjenk & Chen, 2019; Compton et al., 2006; Cullen, 2018). Lastly, our sample was predominantly White (72%), limiting the generalizability of our findings.

## Conclusions

Our findings suggest that slower motor speed may be a useful indicator of suicide risk in psychiatric populations. Psychiatric patients may benefit from a brief cognitive evaluation of motor speed as an objective measure to help identify those at highest risk for suicide attempt who may need additional monitoring and treatment.

## Supporting information

10.1017/S0033291726103675.sm001 Jia-Richards et al. supplementary material Jia-Richards et al. supplementary material

## Supplementary material

The supplementary material for this article can be found at http://doi.org/10.1017/S0033291726103675.
