# Supplementary material for: Slower motor speed as a predictor of suicide attempts in high-risk youth
Source: Psychol Med. 2026 May 5;56:e125. doi: 10.1017/S0033291726103675 (PMC13161802; doi:10.1017/S0033291726103675)
Supplement: Jia-Richards et al. supplementary material [file S0033291726103675sup001.docx]

**Supplementary Tables and Figures**

**Table of Contents**

[Table S1: Univariate Comparisons Between Included and Excluded Participants 2](#_Toc219452784)

[Table S2. Study Retention Rates by Study Group and Timepoint 4](#_Toc219452785)

[Table S3. Comparing Participants Who Were Lost to Follow-up to Those Who Were Retained 5](#_Toc219452786)

[Table S4. Correlations Between Observed Measures in Factor Analyses 7](#_Toc219452787)

[Table S5. Factor Loadings for Exploratory Factor Analyses with Covariate Matrix Estimated with Complete and Missing Data 8](#_Toc219452788)

[Table S6. Model Fit Indices for Confirmatory Factor Analyses at Follow-up Timepoints 9](#_Toc219452789)

[Table S7. Measurement Invariance Model Fits 10](#_Toc219452790)

[Table S8. Post-hoc Group Comparisons of Neurocognitive Performance for Univariate and Reduced Final Multivariate Models 11](#_Toc219452791)

[Table S9. Pooled Univariate Relationships Between Neurocognitive Tasks and Sociodemographic and Clinical Covariates 12](#_Toc219452792)

[Table S10. Comparing Participants with and without Actual SA or SB at 12 Months 13](#_Toc219452793)

[Table S11. Univariate Relationships Between Making a Suicide Attempt and Sociodemographic and Clinical Covariates 15](#_Toc219452794)

[Table S12. Univariate Logistic Regression Models of Neurocognitive Tasks Predicting Actual SA and SB 16](#_Toc219452795)

[Table S13. Multivariate Logistic Regression Models of Neurocognitive Tasks Predicting Actual Suicide Attempts 17](#_Toc219452796)

[Table S14. Pooled DeLong’s Tests Comparing Logistic Models with and without the D/S-IAT or the MOT 18](#_Toc219452797)

[Table S15. Pooled and Reduced ANCOVA Models Examining the Differences in Performance on Neurocognitive Tasks at Baseline by Group Using Complete Data Only 19](#_Toc219452798)

[Table S16. Cognitive Tasks Predicting Time-to-Onset for Actual Suicide Attempts—Cox Models Using Complete Data Only 20](#_Toc219452799)

[Table S17. Multivariate Logistic Regression Models of Neurocognitive Tasks Predicting Actual Suicide Attempts Using Complete Data Only 21](#_Toc219452800)

[Table S18. Number and Percent of Participants with MOT Mean Latency Scores 1.5 SDs Below the Mean 22](#_Toc219452801)

[Table S19. Pooled and Reduced ANCOVA Models Examining the Differences in Performance on Neurocognitive Tasks at Baseline by Group Excluding Participants with MOT Mean Latency Scores <1.5 SDs From the Mean 23](#_Toc219452802)

[Table S20. Cognitive Tasks Predicting Time-to-Onset for Actual Suicide Attempts—Cox Models Excluding Participants with MOT Mean Latency Scores <1.5 SDs From the Mean 24](#_Toc219452803)

[Table S21. Multivariate Logistic Regression Models of Neurocognitive Tasks Predicting Actual Suicide Attempts Excluding Participants with MOT Mean Latency Scores <1.5 SDs From the Mean 25](#_Toc219452804)

# Table S1: Univariate Comparisons Between Included and Excluded Participants

|  | ***M*(SD) or *n*(%)** | |  |  |  |  |
| --- | --- | --- | --- | --- | --- | --- |
| **Characteristic** | **Did Not Complete Any Neurocognitive Measures**  ***N* = 298** | **Completed Neurocognitive Measure(s)**  ***N* = 101** | **Test** | **Statistic** | ***P*** | ***d*** |
| **Group** |  |  | 𝜒^2^(3.00) |  | 0.012* |  |
| SA | 9(9.1%) | 54(18%) | 𝜒^2^(1.00) | 2.59 | 0.108 | 0.19 |
| SI | 33(33%) | 90(30%) | 𝜒^2^(1.00) | 6.97 | 0.008 | 0.31 |
| PC | 39(39%) | 76(26%) | 𝜒^2^(1.00) | 0.34 | 0.559 | 0.07 |
| HC | 18(18%) | 78(26%) | 𝜒^2^(1.00) | 4.54 | 0.033 | 0.25 |
| **Sociodemographic Characteristics** | | | | | | |
| Sex (Male) | 40(40%) | 116(39%) | 𝜒^2^(1.00) | 0.01 | 0.904 | 0.01 |
| Race (White) | 76(76%) | 213(72%) | 𝜒^2^(1.00) | 0.69 | 0.405 | 0.10 |
| Age | 24.42(3.58) | 24.34(3.71) | *t*(177.94) | 0.18 | 0.856 | 0.02 |
| SES | -0.20(0.87) | -0.16(0.78) | *t*(90.49) | −0.28 | 0.777 | 0.04 |
| LGBTQ | 27(45%) | 91(33%) | 𝜒^2^(1.00) | 2.93 | 0.087 | 0.24 |
| **Psychiatric Characteristics** | | | | | | |
| Mood Disorder | 69(78%) | 209(74%) | 𝜒^2^(1.00) | 0.59 | 0.444 | 0.09 |
| Anxiety Disorder | 37(42%) | 126(45%) | 𝜒^2^(1.00) | 0.21 | 0.645 | 0.06 |
| PTSD | 16(18%) | 64(23%) | 𝜒^2^(1.00) | 0.83 | 0.361 | 0.11 |
| Psychosis | 1(1.1%) | 9(3.2%) | Fisher’s |  | 0.462 | 0.13 |
| SUD | 50(57%) | 136(48%) | 𝜒^2^(1.00) | 1.90 | 0.168 | 0.17 |
| Lifetime SB | 63(66%) | 177(60%) | 𝜒^2^(1.00) | 1.11 | 0.292 | 0.09 |
| **Clinical Measures** | | | | | | |
| Depression (PHQ-9) | 12.34(7.98) | 11.24(8.44) | *t*(164.20) | 1.14 | 0.255 | 0.13 |
| Anxiety (GAD-7) | 10.52(6.19) | 8.92(6.55) | *t*(164.37) | 2.16 | 0.033* | 0.25 |
| Affective Liability (ALS) | 1.43(0.83) | 1.24(0.84) | *t*(145.82) | 1.86 | 0.065 | 0.22 |
| Apathy | 41.72(8.60) | 39.33(7.87) | *t*(114.19) | 2.19 | 0.031* | 0.30 |
| Anhedonia (SHAPS) | 3.41(3.38) | 2.84(2.96) | *t*(103.48) | 1.30 | 0.195 | 0.19 |
| Hopelessness (BHS) | 10.13(2.29) | 9.66(2.33) | *t*(151.50) | 1.70 | 0.091 | 0.20 |
| Insomnia | 12.14(7.62) | 10.72(7.41) | *t*(117.23) | 1.45 | 0.150 | 0.19 |
| PTS Symptoms (PCL) | 33.61(21.89) | 28.59(22.75) | *t*(145.46) | 1.84 | 0.067 | 0.22 |
| Substance Use (DUSI-a) | 22.01(5.14) | 21.35(4.98) | *t*(131.15) | 1.05 | 0.296 | 0.13 |
| Substance Use Problems (DUSI-b) | 27.79(31.15) | 25.41(30.51) | *t*(132.43) | 0.62 | 0.535 | 0.08 |
| Impulsivity (BIS) | 69.08(14.68) | 63.65(13.86) | *t*(117.56) | 2.90 | 0.004** | 0.39 |
| Aggression (BPS) | 0.49(0.17) | 0.47(0.16) | *t*(134.03) | 0.79 | 0.433 | 0.10 |
| Perceived Stress (PSS) | 22.95(7.54) | 21.45(9.17) | *t*(138.13) | 1.45 | 0.150 | 0.17 |
| Somatic Symptoms (PHQ-15) | 8.37(5.56) | 7.66(5.23) | *t*(148.72) | 1.09 | 0.277 | 0.13 |
| **Clinical Factors** |  |  |  |  |  |  |
| Factor 1 | 0.06(1.11) | -0.04(0.98) | *t*(86.08) | 0.64 | 0.524 | 0.10 |
| Factor 2 | 0.16(1.16) | -0.03(0.94) | *t*(82.25) | 1.17 | 0.246 | 0.19 |
| Factor 3 | 0.13(0.96) | -0.09(0.92) | *t*(91.26) | 1.62 | 0.108 | 0.24 |
| Factor 4 | -0.07(0.98) | -0.03(0.96) | *t*(92.09) | −0.29 | 0.769 | 0.04 |
| **Risk and Protective Factors** | | | | | | |
| Childhood Abuse (CTQ) | 60.48(11.92) | 57.83(10.60) | *t*(135.40) | 1.89 | 0.061 | 0.24 |
| Social Support (MSPSS) | 5.31(1.29) | 5.38(1.26) | *t*(117.74) | −0.41 | 0.680 | 0.05 |
| **Suicide Outcomes at 12 Months** | | | | | | |
| SB Outcome | 3(3.0%) | 19(6.4%) | 𝜒^2^(1.00) | 1.68 | 0.195 | 0.15 |
| SA Outcome | 6(5.9%) | 43(14%) | 𝜒^2^(1.00) | 5.05 | 0.025* | 0.26 |
| *Note.* **p* < .05, ***p* < .01, ****p* < .001, *d* = Cohen’s *d*. Frequencies are observed frequencies. SA = suicide attempt group; SI = suicidal ideation group; PC = psychiatric control group; HC = healthy control group. SES = Hollingshed scale; WASI-II = Weschler Abbreviated Scale of Intelligence, 2nd edition; PTSD = Posttraumatic Stress Disorder; SUD = Substance Use Disorder; ALS = Affective Liability Scale; Apathy = Apathy Evaluation Scale; BHS = Beck Hopelessness Scale; BIS = Barratt Impulsivity Scale; BPS = Buss-Perry Aggression Questionnaire; GAD-7 = Generalized Anxiety Disorder scale; Insomnia = Insomnia PhenX Toolkit; PHQ-9 = Patient Health Questionnaire; PHQ-15 = Patient Health Questionnaire; PSS = Perceived Stress Scale; PCL = PTSD Checklist for the DSM-5; SHAPS = Snaith-Hamilton Pleasure Scale; DUSI = Drug Use Screening Inventory; CTQ = Childhood Trauma Questionnaire; MSPSS = Multidimensional Scale of Perceived Social Support. | | | | | | |

# Table S2. Study Retention Rates by Study Group and Timepoint

|  | **Follow-Up Assessments—*N*(%)** | | | |
| --- | --- | --- | --- | --- |
| **Group** | **Baseline** | **3 Months** | **6 Months** | **12 Months** |
| HC | 54 | 32(59%) | 24(44%) | 23(43%) |
| PC | 90 | 44(49%) | 28(31%) | 21(23%) |
| SI | 76 | 28(37%) | 19(25%) | 12(16%) |
| SA | 78 | 18(23%) | 14(18%) | 8(10%) |
| **Total** | 298 | 122(41%) | 85(29%) | 64(21%) |
| *Note*. Percentages are calculated by sample size at baseline. HC = healthy control group; PC = psychiatric control group; SI = suicidal ideation group; SA = actual suicide attempt group. | | | | |

# Table S3. Comparing Participants Who Were Lost to Follow-up to Those Who Were Retained

|  | ***M*(SD) or *n*(%)** | |  |  |  |  |
| --- | --- | --- | --- | --- | --- | --- |
| **Characteristic** | **Lost to Follow-Up—Completed 1 Timepoint**  **(*N* = 139, 47%)** | **Retained—Completed >1 Timepoints**  **(*N* = 159, 53%)** | **Test** | **Statistic** | ***p*** | ***d*** |
| **Group** |  |  | Χ²(3.00) | 30.65 | <0.001*** |  |
| SA | 54(39%) | 24(15%) | Χ²(1.00) | 21.66 | <0.001*** | 0.56 |
| SI | 37(27%) | 39(25%) | Χ²(1.00) | 0.17 | 0.680 | 0.05 |
| PC | 36(26%) | 54(34%) | Χ²(1.00) | 2.29 | 0.130 | 0.18 |
| HC | 12(8.6%) | 42(26%) | Χ²(1.00) | 15.81 | <0.001*** | 0.47 |
| **Sociodemographic Characteristics** | | | | | | |
| Sex (Male) | 64(46%) | 52(33%) | Χ²(1.00) | 5.55 | 0.018* | 0.28 |
| Race (White) | 98(71%) | 115(72%) | Χ²(1.00) | 0.06 | 0.802 | 0.03 |
| Age | 23.93(3.66) | 24.70(3.73) | *t*(292.05) | -1.79 | 0.075 | 0.21 |
| SES | -0.46(0.68) | 0.08(0.77) | *t*(278.33) | -6.24 | <0.001*** | 0.74 |
| LGBTQ | 41(33%) | 50(34%) | Χ²(1.00) | 0.01 | 0.932 | 0.01 |
| **Psychiatric Characteristics** | | | | | | |
| Mood Disorder | 108(84%) | 101(66%) | Χ²(1.00) | 12.33 | <0.001*** | 0.43 |
| Anxiety Disorder | 45(35%) | 81(53%) | Χ²(1.00) | 8.91 | 0.003** | 0.36 |
| PTSD | 24(19%) | 40(26%) | Χ²(1.00) | 2.17 | 0.141 | 0.18 |
| Psychosis | 5(3.9%) | 4(2.6%) | Fisher’s | -- | 0.736 | 0.07 |
| SUD | 79(62%) | 57(37%) | Χ²(1.00) | 16.70 | <0.001*** | 0.50 |
| Lifetime SB | 100(72%) | 77(49%) | Χ²(1.00) | 16.54 | <0.001*** | 0.49 |
| **Clinical Measures** | | | | | | |
| Depression (PHQ-9) | 13.63(8.10) | 9.19(8.20) | *t*(288.54) | 4.67 | <0.001*** | 0.54 |
| Anxiety (GAD-7) | 10.76(6.24) | 7.33(6.42) | *t*(289.74) | 4.65 | <0.001*** | 0.54 |
| Affective Liability (ALS) | 1.47(0.77) | 1.04(0.86) | *t*(290.42) | 4.49 | <0.001*** | 0.52 |
| Apathy | 40.85(7.96) | 38.13(7.61) | *t*(252.18) | 2.86 | 0.005** | 0.35 |
| Anhedonia (SHAPS) | 3.19(2.99) | 2.57(2.92) | *t*(252.45) | 1.71 | 0.089 | 0.21 |
| Hopelessness (BHS) | 10.36(2.45) | 9.06(2.03) | *t*(264.87) | 4.94 | <0.001*** | 0.58 |
| Insomnia | 12.61(6.83) | 9.23(7.53) | *t*(266.88) | 3.89 | <0.001*** | 0.47 |
| PTS Symptoms (PCL) | 34.65(21.16) | 23.33(22.84) | *t*(278.77) | 4.32 | <0.001*** | 0.51 |
| Substance Use (DUSI-a) | 22.14(5.40) | 20.67(4.50) | *t*(261.66) | 2.51 | 0.013* | 0.30 |
| Substance Use Problems (DUSI-b) | 34.57(32.16) | 17.48(26.67) | *t*(261.03) | 4.89 | <0.001*** | 0.58 |
| Impulsivity (BIS) | 67.88(12.73) | 60.49(13.87) | *t*(255.88) | 4.53 | <0.001*** | 0.55 |
| Aggression (BPS) | 0.51(0.16) | 0.44(0.15) | *t*(278.73) | 3.52 | 0.001*** | 0.41 |
| Perceived Stress (PSS) | 23.84(8.71) | 19.54(9.11) | *t*(259.58) | 3.96 | <0.001*** | 0.48 |
| Somatic Symptoms (PHQ-15) | 8.71(4.95) | 6.76(5.30) | *t*(292.05) | 3.27 | 0.001** | 0.38 |
| **Clinical Factors** |  |  |  |  |  |  |
| Factor 1 | 0.15(0.95) | -0.18(0.97) | *t*(234.44) | 2.68 | 0.008** | 0.34 |
| Factor 2 | 0.13(1.02) | -0.15(0.87) | *t*(212.07) | 2.32 | 0.021* | 0.30 |
| Factor 3 | 0.06(0.98) | -0.20(0.86) | *t*(215.88) | 2.22 | 0.027* | 0.29 |
| Factor 4 | 0.18(1.07) | -0.19(0.82) | *t*(198.03) | 3.02 | 0.003** | 0.40 |
| **Risk and Protective Factors** | | | | | | |
| Childhood Abuse (CTQ) | 59.35(11.49) | 56.57(9.66) | *t*(256.59) | 2.20 | 0.028* | 0.26 |
| Social Support (MSPSS) | 5.28(1.13) | 5.46(1.35) | *t*(269.83) | -1.20 | 0.231 | 0.14 |
| **Suicide Outcomes at 12 Months** | | | | | | |
| SB Outcome | 8(5.8%) | 11(6.9%) | Χ²(1.00) | 0.17 | 0.682 | 0.05 |
| SA Outcome | 15(11%) | 28(18%) | Χ²(1.00) | 2.79 | 0.095 | 0.19 |
| **Neurocognitive Tasks** | | | | | | |
| D/S-IAT | -0.28(0.41) | -0.28(0.42) | *t*(246.66) | 0.07 | 0.943 | 0.01 |
| Stroop (Suicide) | 6.21(122.48) | 1.02(163.58) | *t*(237.87) | 0.28 | 0.777 | 0.04 |
| MOT Mean Latency (pct) | 0.58(0.30) | 0.46(0.29) | *t*(272.07) | 3.39 | 0.001** | 0.40 |
| OTS Latency to Correct (pct) | 0.53(0.28) | 0.56(0.28) | *t*(266.31) | -0.78 | 0.437 | 0.09 |
| SST SSRT (pct) | 0.56(0.30) | 0.51(0.28) | *t*(265.81) | 1.43 | 0.153 | 0.17 |
| SWM Between Errors (pct) | 0.61(0.26) | 0.53(0.29) | *t*(280.10) | 2.22 | 0.027* | 0.25 |
| *Note.* **p* < .05, ***p* < .01, ****p* < .001, *d* = Cohen’s *d*. Frequencies are observed frequencies. Tasks marked with pct were converted to percentile scores. SA = suicide attempt group; SI = suicidal ideation group; PC = psychiatric control group; HC = healthy control group. SES = Hollingshed scale; WASI-II = Weschler Abbreviated Scale of Intelligence, 2nd edition; PTSD = Posttraumatic Stress Disorder; SUD = Substance Use Disorder; ALS = Affective Liability Scale; Apathy = Apathy Evaluation Scale; BHS = Beck Hopelessness Scale; BIS = Barratt Impulsivity Scale; BPS = Buss-Perry Aggression Questionnaire; GAD-7 = Generalized Anxiety Disorder scale; Insomnia = Insomnia PhenX Toolkit; PHQ-9 = Patient Health Questionnaire; PHQ-15 = Patient Health Questionnaire; PSS = Perceived Stress Scale; PCL = PTSD Checklist for the DSM-5; SHAPS = Snaith-Hamilton Pleasure Scale; DUSI = Drug Use Screening Inventory; CTQ = Childhood Trauma Questionnaire; MSPSS = Multidimensional Scale of Perceived Social Support; D/S IAT = Death/Suicide Implicit Association Test; MOT = Motor Screening Task; OTS = One Touch Stocking task; SST = Stop Signal Task; SSRT = Stop Signal Reaction Time; SWM = Spatial Working Memory Task. | | | | | | |

# Table S4. Correlations Between Observed Measures in Factor Analyses

| **Measure** | **1** | **2** | **3** | **4** | **5** | **6** | **7** | **8** | **9** | **10** | **11** | **12** | **13** |
| --- | --- | --- | --- | --- | --- | --- | --- | --- | --- | --- | --- | --- | --- |
| **1.** Affective Liability (ALS) | -- |  |  |  |  |  |  |  |  |  |  |  |  |
| **2.** Apathy | .44*** | -- |  |  |  |  |  |  |  |  |  |  |  |
| **3.** Hopelessness (BHS) | .46*** | .23*** | -- |  |  |  |  |  |  |  |  |  |  |
| **4.** Impulsivity (BIS) | .65*** | .50*** | .32*** | -- |  |  |  |  |  |  |  |  |  |
| **5.** Aggression (BPS) | .72*** | .25*** | .46*** | .55*** | -- |  |  |  |  |  |  |  |  |
| **6.** Anxiety (GAD-7) | .74*** | .56*** | .35*** | .59*** | .56*** | -- |  |  |  |  |  |  |  |
| **7.** Insomnia (PhenX) | .71*** | .45*** | .38*** | .59*** | .54*** | .68*** | -- |  |  |  |  |  |  |
| **8.** Depression (PHQ-9) | .73*** | .67*** | .39*** | .67*** | .53*** | .86*** | .71*** | -- |  |  |  |  |  |
| **9.** Somatic Symptoms (PHQ-15) | .69*** | .47*** | .29*** | .57*** | .47*** | .74*** | .67*** | .75*** | -- |  |  |  |  |
| **10.** Perceived Stress (PSS) | .77*** | .60*** | .41*** | .63*** | .61*** | .77*** | .69*** | .81*** | .63*** | -- |  |  |  |
| **11.** PTS Symptoms (PCL) | .72*** | .52*** | .39*** | .57*** | .58*** | .74*** | .65*** | .73*** | .64*** | .70*** | -- |  |  |
| **12.** Anhedonia (SHAPS) | .34*** | .50*** | .19** | .44*** | .26*** | .42*** | .36*** | .51*** | .35*** | .47*** | .40*** | -- |  |
| **13.** Substance Use Frequency (DUSI-a) | .36*** | .20*** | .16** | .37*** | .38*** | .37*** | .40*** | .37*** | .33*** | .37*** | .42*** | .17** | -- |
| **14.** Substance Use Problems (DUSI-b) | .44*** | .22*** | .33*** | .53*** | .43*** | .35*** | .41*** | .39*** | .31*** | .39*** | .48*** | .29*** | .62*** |
| *Note.* ***p* < .01, ****p* < .001. ALS = Affective Liability Scale; Apathy = Apathy Evaluation Scale; BHS = Beck Hopelessness Scale; BIS = Barratt Impulsivity Scale; BPS = Buss-Perry Aggression Questionnaire; GAD-7 = Generalized Anxiety Disorder scale; Insomnia = Insomnia PhenX Toolkit; PHQ-9 = Patient Health Questionnaire 9; PHQ-15 = Patient Health Questionnaire 15; PSS = Perceived Stress Scale; PCL = PTSD Checklist for the DSM-5; SHAPS = Snaith-Hamilton Pleasure Scale; DUSI = Drug Use Screening Inventory. | | | | | | | | | | | | | |

# Table S5. Factor Loadings for Exploratory Factor Analyses with Covariate Matrix Estimated with Complete and Missing Data

|  | **Complete Cases Only** | | | | **With Missing Data** | | | |
| --- | --- | --- | --- | --- | --- | --- | --- | --- |
| **Measure** | **Factor 1** | **Factor 2** | **Factor 3** | **Factor 4** | **Factor 1** | **Factor 2** | **Factor 3** | **Factor 4** |
| Depression (PHQ-9) | **0.685** | 0.568 | 0.239 | 0.200 | **0.702** | 0.547 | 0.239 | 0.193 |
| Anxiety (GAD-7) | **0.753** | 0.399 | 0.264 | 0.152 | **0.774** | 0.360 | 0.269 | 0.147 |
| Affective Lability (ALS) | **0.618** | 0.254 | 0.553 | 0.240 | 0.588 | 0.225 | **0.601** | 0.228 |
| Apathy | 0.249 | **0.761** | 0.138 | 0.014 | 0.258 | **0.765** | 0.119 | 0.026 |
| Anhedonia (SHAPS) | 0.195 | **0.564** | 0.072 | 0.070 | 0.185 | **0.553** | 0.102 | 0.095 |
| Hopelessness (BHS) | 0.175 | 0.102 | **0.425** | 0.161 | 0.163 | 0.125 | **0.441** | 0.185 |
| Insomnia | **0.624** | 0.283 | 0.351 | 0.191 | **0.587** | 0.279 | 0.378 | 0.213 |
| PTS Symptoms (PCL) | **0.662** | 0.315 | 0.272 | 0.300 | **0.630** | 0.263 | 0.347 | 0.310 |
| Substance Use (DUSI-a) | 0.292 | 0.019 | 0.198 | **0.532** | 0.271 | 0.013 | 0.173 | **0.574** |
| Substance Use Problems (DUSI-b) | 0.093 | 0.143 | 0.246 | **0.952** | 0.079 | 0.133 | 0.274 | **0.947** |
| Impulsivity (BIS) | 0.294 | **0.486** | 0.441 | 0.329 | 0.294 | **0.479** | 0.461 | 0.341 |
| Aggression (BPS) | 0.343 | 0.121 | **0.651** | 0.264 | 0.345 | 0.102 | **0.664** | 0.245 |
| Perceived Stress (PSS) | **0.582** | 0.461 | 0.438 | 0.157 | **0.580** | 0.457 | 0.452 | 0.146 |
| Somatic Symptoms (PHQ-15) | **0.720** | 0.215 | 0.253 | 0.143 | **0.710** | 0.214 | 0.227 | 0.144 |
| **Eigenvalue** | 3.531 | 2.165 | 1.808 | 1.691 | 3.432 | 2.048 | 1.987 | 1.740 |
| *Note****.*** Bold factor loadings show which factor each measure loads onto the strongest. For analyses including missing data, the covariance matrix was estimated using Full Information Maximum Likelihood (FIML). ALS = Affective Liability Scale; Apathy = Apathy Evaluation Scale; BHS = Beck Hopelessness Scale; BIS = Barratt Impulsivity Scale; BPS = Buss-Perry Aggression Questionnaire; GAD-7 = Generalized Anxiety Disorder scale; Insomnia = Insomnia PhenX Toolkit; PHQ-9 = Patient Health Questionnaire; PHQ-15 = Patient Health Questionnaire; PSS = Perceived Stress Scale; PCL = PTSD Checklist for the DSM-5; SHAPS = Snaith-Hamilton Pleasure Scale; DUSI = Drug Use Screening Inventory. | | | | | | | | |

# Table S6. Model Fit Indices for Confirmatory Factor Analyses at Follow-up Timepoints

| **Timepoint** | **χ^2^(71)** | ***p*** | **CFI/TLI** | **RMSEA (90% CI)** | **SRMR** |
| --- | --- | --- | --- | --- | --- |
| 3-month follow-up | 200.55 | < 0.001* | 0.897/0.868 | 0.113(0.095, 0.132) | 0.062 |
| 6-month follow-up | 159.80 | < 0.001* | 0.889/0.857 | 0.114(0.090, 0.090) | 0.061 |
| 12-month follow-up | 158.24 | < 0.001* | 0.875/0.840 | 0.133(0.106, 0.161) | 0.073 |
| *Note.* **p* < 0.001. CFI = Comparative Fit Index; TLI = Tucker Lewis Index; RMSEA = Root Mean Square Error of Approximation; SRMR = Standardized Root Mean Square Residual. | | | | | |

# Table S7. Measurement Invariance Model Fits

| **Model** | **df** | **χ^2^** | ***p*** | **AIC** | **BIC** | **CFI/TLI** | **RMSEA (90% CI)** | **ΔRMSEA** | **Δχ^2^** | ***p*** |
| --- | --- | --- | --- | --- | --- | --- | --- | --- | --- | --- |
| Configural | 284 | 843.70 | < 0.001* | 20313.26 | 21187.34 | 0.910/0.885 | 0.106(0.098 , 0.114) |  |  |  |
| Metric | 314 | 869.97 | < 0.001* | 20279.53 | 21017.03 | 0.911/0.896 | 0.101(0.093, 0.108) | 0.005 | 26.27 | 0.661 |
| Scalar | 344 | 886.87 | < 0.001* | 20236.43 | 20837.36 | 0.913/0.908 | 0.095(0.087, 0.103) | 0.006 | 16.90 | 0.974 |
| Residual | 386 | 1071.57 | < 0.001* | 20337.12 | 20746.85 | 0.864/0.878 | 0.101(0.094, 0.108) | 0.006 | 184.69 | < 0.001* |
| *Note.* **p* < 0.001. AIC = Akaike Information Criterion; BIC = Bayesian information criterion; CFI = Comparative Fit Index; TLI = Tucker Lewis Index; RMSEA = Root Mean Square Error of Approximation. | | | | | | | | | | |

# Table S8. Post-hoc Group Comparisons of Neurocognitive Performance for Univariate and Reduced Final Multivariate Models

| **Univariate** | | | | **Reduced Multivariate** | | | |
| --- | --- | --- | --- | --- | --- | --- | --- |
| **Contrast** | **Est. Diff (SE)** | ***d* (95% CI)** | ***p*** | **Contrast** | **Est. Diff (SE)** | ***d* (95% CI)** | ***p*** |
| **D/S-IAT** | | | | **D/S-IAT** | | | |
| PC - HC | 0.19 (0.07) | 0.48 (0.12, 0.84) | 0.040* | PC - HC | 0.39(0.18) | 0.41(0.79, 0.04) | 0.131 |
| SA - HC | 0.38 (0.08) | 0.95 (0.57, 1.34) | <0.001*** | SA - HC | 0.83(0.20) | 0.88(1.32, 0.44) | <0.001*** |
| SA - PC | 0.19 (0.07) | 0.47 (0.13, 0.81) | 0.034* | SA - PC | 0.44(0.17) | 0.47(0.83, 0.11) | 0.048* |
| SI - HC | 0.22 (0.08) | 0.54 (0.17, 0.92) | 0.023* | SI - HC | 0.24(0.21) | 0.25(0.69, −0.18) | 0.661 |
| SI - PC | 0.02 (0.07) | 0.06 (-0.27, 0.39) | 0.986 | SI - PC | 0.15(0.17) | 0.16(0.20, −0.52) | 0.811 |
| SI - SA | 0.16 (0.07) | 0.41 (0.05, 0.77) | 0.106 | SI - SA | 0.59(0.18) | 0.63(1.00, 0.25) | 0.005** |
| **MOT Mean Latency (pct)** | | | | **MOT Mean Latency (pct)** | | | |
| PC - HC | 0.10 (0.05) | 0.32 (-0.03, 0.67) | 0.260 | PC - HC | 0.23(0.17) | 0.24(0.59, −0.11) | 0.536 |
| SA - HC | 0.18 (0.05) | 0.61 (0.25, 0.96) | 0.004** | SA - HC | 0.39(0.18) | 0.40(0.78, 0.03) | 0.141 |
| SA - PC | 0.08 (0.05) | 0.28 (-0.03, 0.60) | 0.295 | SA - PC | 0.16(0.16) | 0.16(0.49, −0.16) | 0.754 |
| SI - HC | 0.18 (0.05) | 0.61 (0.25, 0.96) | 0.005** | SI - HC | 0.52(0.17) | 0.54(0.90, 0.18) | 0.016* |
| SI - PC | 0.08 (0.05) | 0.28 (-0.04, 0.60) | 0.304 | SI - PC | 0.29(0.16) | 0.30(0.62, −0.02) | 0.251 |
| SI - SA | 0.00 (0.05) | 0.00 (-0.32, 0.32) | 1.000 | SI - SA | 0.13(0.16) | 0.14(0.19, −0.47) | 0.847 |
| **OTS Latency to Correct (pct)** | | | | **OTS Latency to Correct (pct)** | | | |
| PC - HC | 0.08 (0.05) | 0.31 (-0.04, 0.66) | 0.306 | PC - HC | 0.27(0.18) | 0.28(0.64, −0.08) | 0.416 |
| SA - HC | 0.01 (0.05) | 0.02 (-0.38, 0.33) | 0.999 | SA - HC | 0.00(0.19) | 0.00(0.39, −0.40) | 1.000 |
| SA - PC | 0.09 (0.04) | 0.33 (0.01, 0.65) | 0.173 | SA - PC | 0.28(0.16) | 0.28(0.05, −0.62) | 0.330 |
| SI - HC | 0.14 (0.05) | 0.50 (0.14, 0.86) | 0.031* | SI - HC | 0.44(0.18) | 0.46(0.83, 0.08) | 0.076 |
| SI - PC | 0.05 (0.05) | 0.19 (-0.13, 0.52) | 0.633 | SI - PC | 0.17(0.16) | 0.17(0.50, −0.15) | 0.713 |
| SI - SA | 0.15 (0.05) | 0.53 (0.19, 0.86) | 0.010** | SA - SI | 0.44(0.17) | 0.46(0.80, 0.11) | 0.043* |
| *Note.* **p* < .05, ***p* < .01, ****p* < .001. Results are pooled for multivariate models. Contrasts used estimated marginal means with Tukey-adjusted *p* values. Tasks marked with pct were converted to percentile scores. HC = Heathy Control group; PC = Psychiatric Control group; SI = Suicidal Ideation group; SA = Suicide Attempt group; D/S IAT = Death/Suicide Implicit Association Test; MOT = Motor Screening Task; OTS = One Touch Stocking task. | | | | | | | |

# Table S9. Pooled Univariate Relationships Between Neurocognitive Tasks and Sociodemographic and Clinical Covariates

|  | **D/S-IAT** | | | | | **MOT Mean Latency (pct)** | | | | | **OTS Mean Latency to Correct (pct)** | | | | |
| --- | --- | --- | --- | --- | --- | --- | --- | --- | --- | --- | --- | --- | --- | --- | --- |
| **Parameter** | **β(SE)** | ***t*** | ***df*** | ***p*** | ***R^2^*** | **β(SE)** | ***t*** | ***df*** | ***p*** | ***R^2^*** | **β(SE)** | ***t*** | ***df*** | ***p*** | ***R^2^*** |
| IQ (WASI-II) | 0.03(0.06) | 0.47 | 186.53 | 0.640 | 0.00 | -0.25(0.06) | -4.17 | 146.37 | 0.000* | 0.07 | 0.11(0.06) | 1.83 | 213.65 | 0.069 | 0.01 |
| Age | 0.02(0.07) | 0.26 | 246.02 | 0.798 | 0.00 | 0.09(0.06) | 1.46 | 278.02 | 0.147 | 0.01 | 0.20(0.06) | 3.35 | 271.02 | **0.001*** | 0.04 |
| Sex (Male) | -0.22(0.13) | -1.70 | 246.02 | 0.090 | 0.01 | 0.18(0.12) | 1.52 | 278.02 | 0.131 | 0.01 | 0.08(0.13) | 0.64 | 271.02 | 0.523 | 0.00 |
| Race (White) | -0.03(0.14) | -0.21 | 245.02 | 0.831 | 0.00 | -0.07(0.13) | -0.51 | 276.43 | 0.614 | 0.00 | -0.05(0.14) | -0.38 | 261.73 | 0.706 | 0.00 |
| SES | -0.10(0.07) | -1.61 | 226.31 | 0.108 | 0.01 | -0.19(0.06) | -3.01 | 226.69 | 0.003* | 0.03 | 0.04(0.07) | 0.59 | 201.74 | 0.553 | 0.00 |
| LGBTQ | 0.34(0.15) | 2.29 | 139.01 | 0.024 | 0.02 | -0.07(0.13) | -0.58 | 237.61 | 0.562 | 0.00 | -0.03(0.13) | -0.20 | 227.36 | 0.844 | 0.00 |
| Factor 1 | 0.17(0.06) | 2.96 | 208.15 | 0.003* | 0.03 | 0.18(0.05) | 3.26 | 217.27 | 0.001* | 0.04 | 0.09(0.06) | 1.70 | 221.66 | 0.091 | 0.01 |
| Factor 2 | 0.26(0.06) | 4.73 | 203.62 | 0.000* | 0.08 | 0.02(0.05) | 0.46 | 209.13 | 0.646 | 0.00 | -0.04(0.06) | -0.68 | 177.18 | 0.499 | 0.00 |
| Factor 3 | 0.07(0.06) | 1.10 | 205.88 | 0.271 | 0.00 | 0.14(0.06) | 2.57 | 213.45 | 0.011 | 0.02 | 0.00(0.06) | -0.07 | 199.19 | 0.943 | 0.00 |
| Factor 4 | -0.01(0.05) | -0.21 | 200.80 | 0.836 | 0.00 | 0.11(0.05) | 2.39 | 215.06 | 0.018 | 0.02 | 0.07(0.05) | 1.41 | 215.06 | 0.160 | 0.01 |
| Mood Disorder | 0.56(0.14) | 3.93 | 206.24 | 0.000* | 0.06 | 0.39(0.14) | 2.90 | 242.11 | 0.004* | 0.03 | 0.25(0.14) | 1.78 | 237.37 | 0.077 | 0.01 |
| Anxiety Disorder | 0.17(0.13) | 1.33 | 200.69 | 0.187 | 0.01 | 0.00(0.12) | 0.03 | 251.79 | 0.977 | 0.00 | 0.29(0.12) | 2.36 | 229.04 | 0.019 | 0.02 |
| PTSD | 0.33(0.15) | 2.17 | 209.97 | 0.031 | 0.02 | -0.06(0.15) | -0.37 | 223.47 | 0.710 | 0.00 | 0.40(0.15) | 2.70 | 240.98 | 0.007* | 0.03 |
| Psychosis | -0.17(0.36) | -0.47 | 227.44 | 0.642 | 0.00 | 0.18(0.34) | 0.54 | 257.14 | 0.588 | 0.00 | 0.04(0.35) | 0.12 | 220.05 | 0.904 | 0.00 |
| SUD | 0.12(0.13) | 0.89 | 214.22 | 0.374 | 0.00 | 0.37(0.12) | 3.12 | 251.13 | 0.002* | 0.03 | 0.26(0.12) | 2.10 | 237.12 | 0.037 | 0.02 |
| Childhood Trauma (CTQ) | 0.06(0.06) | 0.94 | 241.30 | 0.348 | 0.00 | 0.08(0.06) | 1.35 | 229.07 | 0.178 | 0.01 | 0.10(0.06) | 1.62 | 252.79 | 0.106 | 0.01 |
| Social Support (MSPSS) | -0.22(0.06) | -3.46 | 218.08 | 0.001* | 0.05 | -0.17(0.06) | -2.84 | 249.63 | 0.005* | 0.03 | -0.07(0.06) | -1.02 | 212.73 | 0.309 | 0.00 |
| *Note*. **p* < .0083. Standardized betas. Tasks marked with pct were converted to percentile scores. WASI-II = Weschler Abbreviated Scale of Intelligence, 2nd edition; D/S IAT = Death/Suicide Implicit Association Test; MOT = Motor Screening Task; OTS = One Touch Stocking task; SES = Hollingshed scale; LGBTQ = Lesbian, Gay, Bisexual, Trans, and Queer identities; PTSD = Posttraumatic Stress Disorder; SUD = Substance Use Disorder; CTQ = Childhood Trauma Questionnaire; MSPSS = Multidimensional Scale of Perceived Social Support. | | | | | | | | | | | | | | | |

# Table S10. Comparing Participants with and without Actual SA or SB at 12 Months

|  |  | **Actual Suicide Attempt** | | | | | | **Suicidal Behaviors** | | | | | |
| --- | --- | --- | --- | --- | --- | --- | --- | --- | --- | --- | --- | --- | --- |
|  |  | ***M*(SD) or *n*(%)** | |  |  |  |  | ***M*(SD) or *n*(%)** | |  |  |  |  |
| **Measures** | ***N*** | **No**  *n* = 225 | **Yes**  *n* = 19 | **Test** | **Value** | ***p*** | ***d*** | **No**  *n* = 201 | **Yes**  *n* = 43 | **Test** | **Value** | ***p*** | ***d*** |
| **Group** | 244 |  |  | 𝜒2(2.00) | 17.48 | 0.002** |  |  |  | 𝜒2(2.00) | 15.66 | <0.001*** |  |
| SA |  | 64(28%) | 14(74%) | 𝜒2(1.00) | 14.47 | 0.001** | 0.58 | 56(28%) | 22(51%) | 𝜒2(1.00) | 7.80 | 0.005** | 0.42 |
| SI |  | 72(32%) | 4(21%) | 𝜒2(1.00) | 0.54 | 0.464 | 0.14 | 60(30%) | 16(37%) | 𝜒2(1.00) | 0.58 | 0.445 | 0.13 |
| PC |  | 89(40%) | 1(5.3%) | 𝜒2(1.00) | 7.44 | 0.006** | 0.40 | 85(42%) | 5(12%) | 𝜒2(1.00) | 13.02 | 0.003 | 0.52 |
| **Sociodemographic Characteristics** | | | |  |  |  |  |  |  |  |  |  |  |
| Sex (Male) | 244 | 85(38%) | 7(37%) | 𝜒2(1.00) | 0.01 | 0.936 | 0.02 | 74(37%) | 18(42%) | 𝜒2(1.00) | 0.38 | 0.536 | 0.10 |
| Race (White) | 243 | 156(70%) | 15(79%) | 𝜒2(1.00) | 0.73 | 0.394 | 0.20 | 142(71%) | 29(67%) | 𝜒2(1.00) | 0.21 | 0.643 | 0.08 |
| Age | 244 | -0.11(0.99) | -0.37(1.08) | *t*(20.62) | 0.99 | 0.333 | 0.26 | -0.12(0.98) | -0.18(1.09) | t(57.42) | 0.29 | 0.774 | 0.05 |
| SES score | 230 | -0.24(0.95) | -0.92(0.81) | *t*(21.15) | 3.38 | 0.003** | 0.73 | -0.29(0.93) | -0.28(1.06) | t(54.33) | -0.04 | 0.969 | 0.01 |
| LGBTQ | 220 | 75(37%) | 10(63%) | 𝜒2(1.00) | 4.14 | 0.042* | 0.53 | 67(37%) | 18(49%) | 𝜒2(1.00) | 1.88 | 0.170 | 0.25 |
| **Psychiatric Characteristics** | | | |  |  |  |  |  |  |  |  |  |  |
| IQ (WASI-II) | 195 | -0.22(1.07) | -0.56(0.58) | *t*(20.52) | 1.95 | 0.066 | 0.33 | -0.26(1.08) | -0.17(0.89) | t(55.35) | -0.51 | 0.613 | 0.08 |
| Mood Disorder | 230 | 190(90%) | 19(100%) |  |  | 0.230 | 0.35 | 168(90%) | 41(95%) |  |  | 0.381 | 0.19 |
| Anxiety Disorder | 230 | 115(55%) | 11(58%) | 𝜒2(1.00) | 0.08 | 0.776 | 0.07 | 101(54%) | 25(58%) | 𝜒2(1.00) | 0.24 | 0.624 | 0.08 |
| PTSD | 230 | 55(26%) | 9(47%) | 𝜒2(1.00) | 3.94 | 0.047* | 0.48 | 43(23%) | 21(49%) | 𝜒2(1.00) | 11.63 | 0.001** | 0.59 |
| Psychosis | 230 | 8(3.8%) | 1(5.3%) |  |  | 0.546 | 0.08 | 6(3.2%) | 3(7.0%) |  |  | 0.375 | 0.19 |
| SUD | 230 | 126(60%) | 9(47%) | 𝜒2(1.00) | 1.10 | 0.295 | 0.25 | 113(60%) | 22(51%) | 𝜒2(1.00) | 1.24 | 0.266 | 0.19 |
| Lifetime SB | 243 | 158(71%) | 19(100%) | 𝜒2(1.00) | 7.69 | 0.006** | 0.67 | 136(68%) | 41(95%) | 𝜒2(1.00) | 13.38 | <0.001*** | 0.63 |
| **Clinical Measures** | | | |  |  |  |  |  |  |  |  |  |  |
| Depression (PHQ-9) | 242 | 13.31(7.64) | 17.03(4.90) | *t*(24.21) | -2.95 | 0.007** | 0.50 | 12.97(7.59) | 16.50(6.55) | t(66.30) | -3.08 | 0.003** | 0.48 |
| Anxiety (GAD-7) | 242 | 10.63(5.86) | 12.22(4.99) | *t*(20.96) | -1.29 | 0.213 | 0.27 | 10.37(5.87) | 12.57(5.18) | t(65.11) | -2.45 | 0.017* | 0.38 |
| Affective Liability (ALS) | 239 | 1.45(0.75) | 1.73(0.78) | *t*(18.36) | -1.44 | 0.166 | 0.37 | 1.44(0.76) | 1.65(0.69) | t(61.61) | -1.73 | 0.088 | 0.28 |
| Apathy | 221 | 40.92(7.50) | 44.43(6.25) | *t*(21.59) | -2.25 | 0.035 | 0.47 | 40.62(7.36) | 44.11(7.34) | t(51.61) | -2.64 | 0.011* | 0.47 |
| Anhedonia (SHAPS) | 219 | 3.05(2.90) | 6.00(3.09) | *t*(19.77) | -3.91 | 0.001** | 1.01 | 3.05(2.91) | 4.45(3.27) | t(48.32) | -2.42 | 0.019* | 0.47 |
| Hopelessness (BHS) | 242 | 9.91(2.36) | 10.39(2.30) | *t*(19.97) | -0.85 | 0.404 | 0.20 | 40.62(7.36) | 44.11(7.34) | t(51.61) | -2.64 | 0.011* | 0.47 |
| Insomnia (PhenX) | 221 | 12.30(7.00) | 15.67(5.09) | *t*(23.14) | -2.60 | 0.016* | 0.49 | 12.40(7.01) | 13.45(6.46) | t(54.45) | -0.89 | 0.378 | 0.15 |
| PTS Symptoms (PCL) | 232 | 33.72(21.09) | 42.67(16.63) | *t*(21.88) | -2.14 | 0.044* | 0.43 | 32.60(20.82) | 42.85(19.28) | t(61.74) | -3.05 | 0.003** | 0.50 |
| Substance Use (DUSI-a) | 238 | 22.02(5.11) | 23.00(6.31) | *t*(17.65) | -0.62 | 0.541 | 0.19 | 22.17(5.27) | 21.73(4.87) | t(61.13) | 0.51 | 0.609 | 0.08 |
| Substance Use Problems (DUSI-b) | 237 | 29.20(31.25) | 42.50(36.01) | *t*(16.68) | -1.44 | 0.169 | 0.42 | 28.76(31.04) | 36.67(34.36) | t(52.71) | -1.35 | 0.184 | 0.25 |
| Impulsivity (BIS) | 216 | 66.49(13.28) | 70.66(11.72) | *t*(21.18) | -1.43 | 0.167 | 0.32 | 66.14(13.01) | 70.30(13.68) | t(48.49) | -1.68 | 0.099 | 0.32 |
| Aggression (BPS) | 239 | 0.51(0.15) | 0.54(0.19) | *t*(17.47) | -0.74 | 0.469 | 0.23 | 0.50(0.14) | 0.53(0.17) | t(52.58) | -0.95 | 0.346 | 0.18 |
| Perceived Stress (PSS) | 218 | 24.15(7.41) | 27.50(3.01) | *t*(39.53) | -3.79 | 0.001** | 0.47 | 23.99(7.47) | 26.57(5.36) | t(68.11) | -2.48 | 0.016* | 0.36 |
| Somatic Symptoms (PHQ-15) | 242 | 8.78(4.83) | 10.61(4.97) | *t*(19.67) | -1.51 | 0.148 | 0.38 | 8.68(4.80) | 10.03(5.03) | t(57.76) | -1.60 | 0.116 | 0.28 |
| **Clinical Factors** |  |  |  |  |  |  |  |  |  |  |  |  |  |
| Factor 1 | 200 | 0.23(1.08) | 0.55(0.94) | *t*(18.62) | -1.28 | 0.216 | 0.30 | 0.20(1.10) | 0.52(0.91) | t(49.69) | -1.71 | 0.093 | 0.29 |
| Factor 2 | 200 | 0.12(1.15) | 0.86(1.42) | *t*(16.76) | -2.03 | 0.059 | 0.63 | 0.09(1.14) | 0.65(1.30) | t(40.68) | -2.29 | 0.027* | 0.48 |
| Factor 3 | 200 | 0.09(1.05) | -0.07(1.31) | *t*(16.73) | 0.48 | 0.634 | 0.15 | 0.10(1.05) | -0.03(1.18) | t(40.90) | 0.62 | 0.542 | 0.13 |
| Factor 4 | 200 | 0.00(1.33) | 0.58(1.63) | *t*(16.78) | -1.39 | 0.183 | 0.43 | -0.04(1.32) | 0.51(1.47) | t(41.16) | -1.98 | 0.055 | 0.41 |
| **Risk and Protective Factors** |  |  |  |  |  |  |  |  |  |  |  |  |  |
| Childhood Abuse (CTQ) | 238 | 0.16(1.09) | 0.54(1.40) | *t*(18.73) | -1.11 | 0.283 | 0.33 | 0.15(1.08) | 0.37(1.28) | t(52.48) | -1.03 | 0.307 | 0.20 |
| Social Support (MSPSS) | 220 | -0.21(0.91) | -1.27(1.02) | *t*(19.52) | 4.27 | <0.001*** | 1.15 | -0.21(0.92) | -0.77(1.06) | t(45.75) | 2.96 | 0.005** | 0.60 |
| **Neurocognitive Tasks** |  |  |  |  |  |  |  |  |  |  |  |  |  |
| D/S-IAT | 201 | 0.10(1.01) | 0.63(0.97) | *t*(17.94) | -2.13 | 0.048 | 0.53 | 0.11(1.03) | 0.31(0.91) | t(51.60) | -1.14 | 0.258 | 0.20 |
| Stroop (Suicide) | 196 | -0.03(1.16) | 0.09(0.48) | *t*(33.60) | -0.85 | 0.404 | 0.11 | -0.02(1.19) | -0.02(0.66) | t(85.98) | -0.02 | 0.984 | 0.00 |
| MOT Mean Latency (pct) | 230 | 0.15(1.02) | 0.66(0.87) | *t*(21.17) | -2.36 | 0.028* | 0.51 | 0.14(1.02) | 0.42(0.94) | t(62.41) | -1.74 | 0.087 | 0.28 |
| OTS Latency to Correct (pct) | 223 | 0.11(1.02) | -0.22(1.13) | *t*(19.50) | 1.20 | 0.243 | 0.32 | 0.09(1.02) | 0.05(1.07) | t(53.80) | 0.21 | 0.835 | 0.04 |
| SST SSRT (pct) | 227 | 0.02(1.04) | 0.34(0.96) | *t*(20.54) | -1.34 | 0.194 | 0.31 | 0.00(1.04) | 0.26(0.99) | t(58.95) | -1.55 | 0.127 | 0.26 |
| SWM Between Errors (pct) | 230 | 0.16(0.94) | -0.23(0.93) | *t*(20.09) | 1.71 | 0.102 | 0.42 | 0.16(0.94) | -0.02(0.94) | t(58.55) | 1.11 | 0.271 | 0.19 |
| *Note.* **p* < .05, ***p* < .01, ****p* < .001; *d* = Cohen’s *d*. Frequencies are observed frequencies. Tasks marked with pct were converted to percentile scores. SA = suicide attempt group; SI = suicidal ideation group; PC = psychiatric control group; HC = healthy control group. SES = Hollingshed scale; WASI-II = Weschler Abbreviated Scale of Intelligence, 2nd edition; PTSD = Posttraumatic Stress Disorder; SUD = Substance Use Disorder; ALS = Affective Liability Scale; Apathy = Apathy Evaluation Scale; BHS = Beck Hopelessness Scale; BIS = Barratt Impulsivity Scale; BPS = Buss-Perry Aggression Questionnaire; GAD-7 = Generalized Anxiety Disorder scale; Insomnia = Insomnia PhenX Toolkit; PHQ-9 = Patient Health Questionnaire; PHQ-15 = Patient Health Questionnaire; PSS = Perceived Stress Scale; PCL = PTSD Checklist for the DSM-5; SHAPS = Snaith-Hamilton Pleasure Scale; DUSI = Drug Use Screening Inventory; CTQ = Childhood Trauma Questionnaire; MSPSS = Multidimensional Scale of Perceived Social Support. | | | | | | | | | | | | | |

# Table S11. Univariate Relationships Between Making a Suicide Attempt and Sociodemographic and Clinical Covariates

| **Measure** | **HR(SE)** | **95% CI** | ***p*** |
| --- | --- | --- | --- |
| Age | 0.78(0.19) | 0.49, 1.25 | 0.297 |
| Sex (Male) | 1.00(0.54) | 0.39, 2.53 | 0.999 |
| Race (White) | 1.46(0.97) | 0.50, 4.28 | 0.493 |
| SES | 0.47(0.14) | 0.26, 0.82 | 0.009** |
| LGBTQ | 2.97(1.80) | 1.09, 8.13 | 0.034* |
| Factor 1 | 1.19(0.27) | 0.77, 1.82 | 0.434 |
| Factor 2 | 1.58(0.31) | 1.09, 2.31 | 0.017* |
| Factor 3 | 0.96(0.22) | 0.61, 1.49 | 0.840 |
| Factor 4 | 1.28(0.21) | 0.93, 1.75 | 0.130 |
| Mood Disorder | 4.43(20.12) | 0.25, 79.10 | 0.312 |
| Anxiety Disorder | 1.04(0.55) | 0.42, 2.58 | 0.938 |
| PTSD | 2.51(1.32) | 1.02, 6.18 | 0.046* |
| Psychosis | 2.01(2.73) | 0.36, 11.08 | 0.425 |
| SUD | 0.63(0.33) | 0.26, 1.55 | 0.314 |
| Childhood Trauma (CTQ) | 1.32(0.25) | 0.92, 1.89 | 0.128 |
| Social Support (MSPSS) | 0.40(0.09) | 0.26, 0.63 | < 0.001*** |
| *Note*. **p* < 0.05, ***p* < 0.01, ****p* < .001. Effects are standardized and pooled. SES = Hollingshed scale; LGBTQ = Lesbian, Gay, Bisexual, Trans, and Queer identities; PTSD = Posttraumatic Stress Disorder; SUD = Substance Use Disorder; CTQ = Childhood Trauma Questionnaire; MSPSS = Multidimensional Scale of Perceived Social Support. | | | |

# Table S12. Univariate Logistic Regression Models of Neurocognitive Tasks Predicting Actual SA and SB

|  | **SA Outcome** | | | | **SB Outcome** | | | |
| --- | --- | --- | --- | --- | --- | --- | --- | --- |
| **Task** | **OR(SE)** | **95% CI** | ***p*** | **AUC** | **OR(SE)** | **95% CI** | ***p*** | **AUC** |
| D/S-IAT | 1.72(0.48) | 1.01, 2.91 | 0.041* | 0.64 | 1.22(0.23) | 0.84, 1.76 | 0.287 | 0.56 |
| Stroop: Suicide Interference | 1.14(0.35) | 0.64, 2.02 | 0.639 | 0.53 | 1.00(0.17) | 0.72, 1.40 | 0.984 | 0.51 |
| MOT Mean Latency (pct) | 1.73(0.49) | 1.01, 2.94 | 0.038* | 0.64 | 1.33(0.24) | 0.94, 1.88 | 0.084 | 0.58 |
| OTS Mean Latency to Correct (pct) | 0.73(0.18) | 0.46, 1.17 | 0.184 | 0.59 | 0.96(0.17) | 0.69, 1.35 | 0.883 | 0.51 |
| SST SSRT (pct) | 1.37(0.36) | 0.84, 2.26 | 0.217 | 0.59 | 1.30(0.23) | 0.92, 1.83 | 0.134 | 0.57 |
| SWM Between Errors (pct) | 0.65(0.18) | 0.39, 1.08 | 0.711 | 0.63 | 0.82(0.15) | 0.57, 1.17 | 0.394 | 0.56 |
| *Note.* ORs are standardized ORs. Tasks marked with pct were converted to percentile scores. SA = actual suicide attempt; SB = suicidal behaviors; D/S IAT = Death/Suicide Implicit Association Test; MOT = Motor Screening Task; OTS = One Touch Stocking task; SST = Stop Signal Task; SSRT = Stop Signal Reaction Time; SWM = Spatial Working Memory Task. | | | | | | | | |

# Table S13. Multivariate Logistic Regression Models of Neurocognitive Tasks Predicting Actual Suicide Attempts

| **Clinical Model** | | | |
| --- | --- | --- | --- |
| **Parameter** | **OR(SE)** | **95% CI** | ***p*** |
| SES | 0.57(0.20) | 0.30, 1.08 | 0.087 |
| Social Support (MSPSS) | 0.40(0.11) | 0.24, 0.68 | 0.001** |
| *Pooled AUC = 0.80, SE = 0.04* | | | |
| **D/S-IAT** | | | |
| **Parameter** | **OR(SE)** | **95% CI** | ***p*** |
| D/S-IAT | 1.61(0.52) | 0.89, 2.93 | 0.116 |
| SES | 0.58(0.22) | 0.29, 1.15 | 0.121 |
| Social Support (MSPSS) | 0.44(0.13) | 0.25, 0.78 | 0.005** |
| *Pooled AUC = 0.79, SE = 0.05* | | | |
| **MOT Mean Latency** | | | |
| **Parameter** | **OR(SE)** | **95% CI** | ***p*** |
| MOT Mean Latency (pct) | 1.60(0.51) | 0.89, 2.88 | 0.119 |
| SES | 0.71(0.25) | 0.37, 1.37 | 0.308 |
| Social Support (MSPSS) | 0.35(0.11) | 0.20, 0.62 | <0.001*** |
| *Pooled AUC = 0.85, SE = 0.03* | | | |
| *Note.* ***p* < 0.01, ****p* < 0.001. ORs are standardized. Tasks marked with pct were converted to percentile scores. SES = Hollingshed scale; MSPSS = Multidimensional Scale of Perceived Social Support. | | | |

# Table S14. Pooled DeLong’s Tests Comparing Logistic Models with and without the D/S-IAT or the MOT

| **Model** | **AUC(SE)** | ***z*(SE)** | ***p*** |
| --- | --- | --- | --- |
| Clinical Model | 0.84(0.07) |  |  |
| Clinical Model + D/S-IAT | 0.84(0.08) | −0.55(0.35) | 0.593 |
| Clinical Model + MOT Mean Latency | 0.90(0.06) | −1.70(0.39) | 0.091 |
| *Note*. D/S IAT = Death/Suicide Implicit Association Test; MOT = Motor Screening Task. | | | |

# Table S15. Pooled and Reduced ANCOVA Models Examining the Differences in Performance on Neurocognitive Tasks at Baseline by Group Using Complete Data Only

| **Parameter** | ***SS*** | ***F*** | ***df*** | **β*(SE)*** | ***p*** | **Cohen's *f*** |
| --- | --- | --- | --- | --- | --- | --- |
| **Model: D/S-IAT**  *R*^2^ = 0.18, *F*(5, 178) = 7.71,  *p* = < 0.001 | | | | | | |
| Group | 11.57 | 4.29 | 3 |  | <0.001*** | 0.09 |
| IQ (WASI-II) | 5.89 | 6.55 | 1 | 0.20(0.08) | 0.011* | 0.05 |
| Factor 2 | 11.17 | 12.41 | 1 | 0.27(0.08) | <0.001*** | 0.07 |
| Residuals | 160.16 |  | 178 |  |  |  |
| **Model: MOT Mean Latency (pct)** *R*^2^ = 0.12, *F*(4, 233) = 8.15, *p* = < 0.001 | | | | | | |
| Group | 10.27 | 3.65 | 3 |  | 0.013* | 0.07 |
| IQ (WASI-II) | 14.24 | 15.17 | 1 | -0.25(0.07) | <0.001*** | 0.06 |
| Residuals | 218.59 |  | 233 |  |  |  |
| **Model: OTS Mean Latency to Correct (pct)** *R*^2^ = 0.08, *F*(6,211) = 3.24, *p* = 0.005 | | | | | | |
| Group | 6.15 | 2.24 | 3 |  | 0.085 | 0.04 |
| IQ (WASI-II) | 1.12 | 1.22 | 1 | 0.07(0.07) | 0.271 | 0.00 |
| Age | 4.13 | 4.51 | 1 | 0.14(0.07) | 0.035* | 0.03 |
| PTSD | 2.67 | 2.91 | 1 | 0.28(0.16) | 0.090 | 0.01 |
| Residuals | 193.39 |  | 211 |  |  |  |
| *Note.* **p* < .05, ***p* < .01, ****p* < .001. Effects are standardized. Tasks marked with pct were converted to percentile scores. Cohen’s *f* = Partial Cohen's *f*. D/S-IAT = Death/Suicide Implicit Association Test; MOT = Motor Screening Task; OTS = One Touch Stocking task; WASI-II = Weschler Abbreviated Scale of Intelligence; MSPSS = Multidimensional Scale of Perceived Social Support; PTSD = Posttraumatic Stress Disorder. | | | | | | |

# Table S16. Cognitive Tasks Predicting Time-to-Onset for Actual Suicide Attempts—Cox Models Using Complete Data Only

| **D/S-IAT** | | | |
| --- | --- | --- | --- |
| **Parameter** | **HR(SE)** | **95% CI** | ***p*** |
| D/S-IAT | 0.96(0.27) | 0.43, 1.48 | 0.875 |
| SES | 0.42(0.14) | 0.14, 0.70 | 0.006** |
| Social Support (MSPSS) | 0.59(0.15) | 0.29, 0.89 | 0.035* |
| **MOT Mean Latency** | | | |
| **Parameter** | **HR(SE)** | **95% CI** | ***p*** |
| MOT Mean Latency (pct) | 2.04(0.69) | 0.69, 3.38 | 0.016* |
| SES | 0.62(0.20) | 0.24, 1.01 | 0.111 |
| Social Support (MSPSS) | 0.60(0.14) | 0.33, 0.88 | 0.030* |
| *Note.* **p* < .05. Estimates are standardized. Tasks marked with pct were converted to percentile scores. D/S-IAT = Death/Suicide Implicit Association Test; MOT = Motor Screening Task; SES = Hollingshed scale; MSPSS = Multidimensional Scale of Perceived Social Support. | | | |

# Table S17. Multivariate Logistic Regression Models of Neurocognitive Tasks Predicting Actual Suicide Attempts Using Complete Data Only

| **D/S-IAT** | | | |
| --- | --- | --- | --- |
| **Parameter** | **OR(SE)** | **95% CI** | ***p*** |
| D/S-IAT | 1.58(0.52) | 0.86, 2.91 | 0.143 |
| SES | 0.54(0.20) | 0.27, 1.07 | 0.078 |
| Social Support (MSPSS) | 0.42(0.13) | 0.24, 0.74 | 0.003** |
| *AUC = 0.80* | | | |
| **MOT Mean Latency** | | | |
| **Parameter** | **OR(SE)** | **95% CI** | ***p*** |
| MOT Mean Latency (pct) | 1.94(0.71) | 0.99, 3.79 | 0.052 |
| SES | 0.74(0.26) | 0.39, 1.42 | 0.372 |
| Social Support (MSPSS) | 0.33(0.10) | 0.18, 0.58 | <0.001*** |
| *AUC = 0.88* | | | |
| *Note.* ***p* < 0.01, ****p* < 0.001. ORs are standardized. Tasks marked with pct were converted to percentile scores. SES = Hollingshed scale; MSPSS = Multidimensional Scale of Perceived Social Support. | | | |

# Table S18. Number and Percent of Participants with MOT Mean Latency Scores 1.5 SDs Below the Mean

|  | **Group** | | | |
| --- | --- | --- | --- | --- |
|  | **SA** | **SI** | **PC** | **HC** |
| N(%) | 2(2.63%) | 6(8.11%) | 7(8.75%) | 6(11.32%) |
| *Note.* SA = actual suicide attempt group; SI = suicidal ideation group; PC = psychiatric control group; HC = healthy control group. | | | | |

# Table S19. Pooled and Reduced ANCOVA Models Examining the Differences in Performance on Neurocognitive Tasks at Baseline by Group Excluding Participants with MOT Mean Latency Scores <1.5 SDs From the Mean

| **Parameter** | ***SS*** | ***F*** | ***df*** | **β*(SE)*** | ***p*** | **Cohen's *f*** |
| --- | --- | --- | --- | --- | --- | --- |
| **Model: D/S-IAT**  *R*^2^ = 0.18, *F*(5, 151.15) = 8.68,  *p* <0.001 | | | | | | |
| Group | 16.36 | 6.00 | 3 |  | <0.001*** | 0.09 |
| IQ (WASI-II) | 3.63 | 3.35 | 1 | 0.13(0.07) | 0.068 | 0.02 |
| Factor 2 | 12.31 | 13.51 | 1 | 0.24(0.07) | <0.001*** | 0.65 |
| Residuals | 189.53 |  | 151.15 |  |  |  |
| **Model: MOT Mean Latency (pct)** *R*^2^ = 0.10, *F*(4, 207.56) = 7.05, *p* <0.001 | | | | | | |
| Group | 7.69 | 3.26 | 3 |  | 0.021* | 0.04 |
| IQ (WASI-II) | 11.54 | 13.11 | 1 | -0.21(0.06) | <0.001*** | 0.06 |
| Residuals | 201.46 | 207.56 |  |  |  |  |
| **Model: OTS Mean Latency to Correct (pct)** *R*^2^ = 0.11, *F*(6, 188.20) = 4.76, *p* <0.001 | | | | | | |
| Group | 9.55 | 3.31 | 3 |  | 0.019* | 0.04 |
| IQ (WASI-II) | 1.47 | 1.16 | 1 | 0.07(0.07) | 0.282 | 0.01 |
| Age | 6.60 | 6.88 | 1 | 0.17(0.06) | 0.009** | 0.03 |
| PTSD | 5.24 | 5.33 | 1 | 0.36(0.16) | 0.021* | 0.02 |
| Residuals | 237.25 |  | 188.20 |  |  |  |
| *Note.* **p* < .05, ***p* < .01, ****p* < .001. Effects are standardized and pooled. Tasks marked with pct were converted to percentile scores. Cohen’s *f* = Partial Cohen's *f*. D/S-IAT = Death/Suicide Implicit Association Test; MOT = Motor Screening Task; OTS = One Touch Stocking task; WASI-II = Weschler Abbreviated Scale of Intelligence; MSPSS = Multidimensional Scale of Perceived Social Support; PTSD = Posttraumatic Stress Disorder. | | | | | | |

# Table S20. Cognitive Tasks Predicting Time-to-Onset for Actual Suicide Attempts—Cox Models Excluding Participants with MOT Mean Latency Scores <1.5 SDs From the Mean

| **D/S-IAT** | | | |
| --- | --- | --- | --- |
| **Parameter** | **HR(SE)** | **95% CI** | ***p*** |
| D/S-IAT | 1.21(0.36) | 0.70, 2.10 | 0.499 |
| SES | 0.60(0.20) | 0.33, 1.12 | 0.109 |
| Social Support (MSPSS) | 0.55(0.15) | 0.33, 0.91 | 0.020* |
| **MOT Mean Latency** | | | |
| **Parameter** | **HR(SE)** | **95% CI** | ***p*** |
| MOT Mean Latency (pct) | 1.61(0.48) | 0.92, 2.80 | 0.095 |
| SES | 0.65(0.19) | 0.38, 1.12 | 0.123 |
| Social Support (MSPSS) | 0.65(0.19) | 0.35, 0.89 | 0.016* |
| *Note.* **p* < .05. Estimates are standardized and pooled. Tasks marked with pct were converted to percentile scores. D/S-IAT = Death/Suicide Implicit Association Test; MOT = Motor Screening Task; SES = Hollingshed scale; MSPSS = Multidimensional Scale of Perceived Social Support. | | | |

# Table S21. Multivariate Logistic Regression Models of Neurocognitive Tasks Predicting Actual Suicide Attempts Excluding Participants with MOT Mean Latency Scores <1.5 SDs From the Mean

| **D/S-IAT** | | | |
| --- | --- | --- | --- |
| **Parameter** | **OR(SE)** | **95% CI** | ***p*** |
| D/S-IAT | 1.77(0.61) | 0.94, 3.33 | 0.076 |
| SES | 0.66(0.25) | 0.33, 1.33 | 0.245 |
| Social Support (MSPSS) | 0.40(0.13) | 0.22, 0.73 | 0.003** |
| *AUC = 0.80, SE = 0.05* | | | |
| **MOT Mean Latency** | | | |
| **Parameter** | **OR(SE)** | **95% CI** | ***p*** |
| MOT Mean Latency (pct) | 1.48(0.50) | 0.80, 2.75 | 0.209 |
| SES | 0.70(0.25) | 0.36, 1.35 | 0.289 |
| Social Support (MSPSS) | 0.36(0.11) | 0.20, 0.63 | <0.001*** |
| *AUC = 0.84, SE = 0.04* | | | |
| *Note.* ***p* < 0.01, ****p* < 0.001. ORs are standardized and pooled. Tasks marked with pct were converted to percentile scores. SES = Hollingshed scale; MSPSS = Multidimensional Scale of Perceived Social Support. | | | |
